# Transcriptome analysis and physiological changes in the leaves of two *Bromus inermis* L. genotypes in response to salt stress

**DOI:** 10.3389/fpls.2023.1313113

**Published:** 2023-12-14

**Authors:** Wenxue Song, Xueqin Gao, Huiping Li, Shuxia Li, Jing Wang, Xing Wang, Tongrui Wang, Yunong Ye, Pengfei Hu, Xiaohong Li, Bingzhe Fu

**Affiliations:** ^1^ College of Forestry and Prataculture, Ningxia University, Yinchuan, Ningxia, China; ^2^ Ningxia Grassland and Animal Husbandry Engineering Technology Research Center, Yinchuan, Ningxia, China; ^3^ Key Laboratory for Model Innovation in Forage Production Efficiency, Ministry of Agriculture and Rural Affairs, Yinchuan, Ningxia, China

**Keywords:** *Bromus inermis* L., salt stress, accessions evaluation, physiological analysis, transcriptome analysis

## Abstract

Soil salinity is a major factor threatening the production of crops around the world. Smooth bromegrass (*Bromus inermis* L.) is a high-quality grass in northern and northwestern China. Currently, selecting and utilizing salt-tolerant genotypes is an important way to mitigate the detrimental effects of salinity on crop productivity. In our research, salt-tolerant and salt-sensitive varieties were selected from 57 accessions based on a comprehensive evaluation of 22 relevant indexes, and their salt-tolerance physiological and molecular mechanisms were further analyzed. Results showed significant differences in salt tolerance between 57 genotypes, with Q25 and Q46 considered to be the most salt-tolerant and salt-sensitive accessions, respectively, compared to other varieties. Under saline conditions, the salt-tolerant genotype Q25 not only maintained significantly higher photosynthetic performance, leaf relative water content (RWC), and proline content but also exhibited obviously lower relative conductivity and malondialdehyde (MDA) content than the salt-sensitive Q46 (*p* < 0.05). The transcriptome sequencing indicated 15,128 differentially expressed genes (DEGs) in Q46, of which 7,885 were upregulated and 7,243 downregulated, and 12,658 DEGs in Q25, of which 6,059 were upregulated and 6,599 downregulated. The Kyoto Encyclopedia of Genes and Genomes (KEGG) analysis showed that the salt response differences between Q25 and Q46 were attributed to the variable expression of genes associated with plant hormone signal transduction and MAPK signaling pathways. Furthermore, a large number of candidate genes, related to salt tolerance, were detected, which involved transcription factors (zinc finger proteins) and accumulation of compatible osmolytes (glutathione *S*-transferases and pyrroline-5-carboxylate reductases), etc. This study offers an important view of the physiological and molecular regulatory mechanisms of salt tolerance in two smooth bromegrass genotypes and lays the foundation for further identification of key genes linked to salt tolerance.

## Introduction

Soil salinity is one of the major factors threatening agricultural production on a global scale (Wang et al., 2020). A high concentration of salt inhibits the growth of plants via osmotic stress and ion imbalance in plant cells (Zhao et al., 2022). What is worse, soil salinization is accelerated by irrational use and exploitation of land, and by 2050, half the world’s arable land will be lost (Zhu, 2016; Albaladejo et al., 2018). If these saline soils are fully utilized for agricultural production, the problems of the demand for food caused by the expanding population and the growth of spendable income can be alleviated. However, as the majority of the crops are sensitive to salinity, it is not feasible to cultivate the varieties currently used in saline–alkaline lands (Van Bezouw et al., 2019; Ganie et al., 2021). It is vital to enhance a plant’s tolerance to salinity so as to support plant growth and yield. Hence, it is necessary to analyze the genes and molecular mechanisms related to salinity responses for the purpose of enhancing the ability to resist salt stress, which would then make significant contributions to the genetic engineering and food production of plants in salt-affected areas.

Currently, the salt tolerance of Gramineae is the focus of many studies (Van Bezouw et al., 2019; Yu et al., 2020; Ganie et al., 2021). Highly saline conditions disturb the homeostasis of water potential and ion distribution, leading to hyperosmotic stress, ion disequilibrium, and oxidative damage, which ultimately threaten plants (Liang et al., 2018; Lv et al., 2019). However, plants have also developed several pathways to respond to high salt stress, including stress-sensing regulatory genes and proteins (Bhattarai et al., 2020). Second messengers, such as Ca^2+^, reactive oxygen species (ROS), and phytohormones were created at a rapid rate in the cytoplasm under salt stress, which progressively decipher and amplify the salt stress signal, enabling a variety of stress signaling receptors found on the cell membrane to rapidly detect changes in the surroundings (Fahad et al., 2015; Steinhorst and Kudla, 2019). These signals modulate downstream transcription factors (TFs) via a cascade response to further alter the transcript levels of a large number of TFs, including WRKY, AP2/ERF, and MYB (Zhao et al., 2020). Then, the expression of a large number of genes, such as *ZFP179* (zinc-finger protein 179) (Sun et al., 2010), *S1CBL10* (calcineurin B-like protein 10) (Egea et al., 2018), *GsPRX9* (a peroxidase gene) (Jin et al., 2019), *SbNHXLP* (a Na^+^/H^+^ antiporter-like protein) (Kumari et al., 2017), and *MsFLS13* (saline–alkaline-induced flavonol synthase gene) (Zhang et al., 2023), are eventually affected, resulting in the development of the salt tolerance in the plants. In addition, many salt tolerance-related transporters or channels, including the plasma membrane Na^+^/H^+^ antiporter (SOS1), have been identified in Gramineae. K^+^ transporter 1 (AKT1), K^+^ uptake transporter (KUP1), Na^+^/H^+^ antiporter (NHX), and high-affinity K^+^ transporter (HKT) have been identified in Gramineae (Zhu, 2016; Bailey-Serres et al., 2019; Gong et al., 2020; Wang et al., 2021).

Smooth bromegrass is considerable forage with excellent feed value, good palatability, and strong adaptability (Li et al., 2022). It is widely cultivated in northern and northwestern China for grazing and sand binding (Li et al., 2022). However, the combination of climate change and agricultural mismanagement exacerbates soil salinization in these areas, limiting its wide use (Wang and Li, 2013; Wang et al., 2021). Li et al. (2022) found that the physiological characteristics, function of the gene, and mechanism of adaptation have undergone changes under salt stress in smooth bromegrass. Hence, it is necessary to explore the physiological and molecular mechanisms of smooth bromegrass response to salt stress, which will make a significant contribution to forage production in saline–alkaline land.

In the research, the evaluation of salt tolerance was performed in 57 smooth bromegrass accessions by analyzing the indexes during the germination and seedling stages. The salt-tolerant variety Q25 and salt-sensitive Q46 were then screened, and their response mechanisms to salt stress were further investigated by transcriptome analysis. Our objective is to gain a broad understanding of the transcriptional expression of genes related to the response to salt stress and to uncover the candidate genes and mechanisms in smooth bromegrass.

## Materials and methods

### Plant materials

For the evaluation of salt tolerance, a total of 57 smooth bromegrass accessions were used. Among them, one genotype was from commercial cultivars, obtained from Inner Mongolia Agricultural University; two accessions were collected from Romania and the United States, obtained from the Institute of Grassland Research; and the others were collected from China (**Supplementary Table 1**).

### Plant growth and treatments

Based on preliminary experiments, the optimal salt concentrations for screening germplasm during the germination and seedling stages were 150 mM and 300 mM, respectively. However, the data from the preliminary experiments were not presented.

In the germination stage, seeds with uniform size were selected from each germplasm, disinfected with 75% ethanol for 30 s, and rinsed six times with distilled water. Fifty sterile seeds were germinated at 25°C/20°C under a light–dark photoperiod of 12/12 h on Petri dishes containing two layers of filter paper moistened with 5 ml of distilled water or 150 mM NaCl solution. Three biological replicates were performed. The filter paper was changed every 2 days, and the number of germinated seeds and moldy seeds was recorded. The indexes were determined when there were no germinated seeds under salt stress (12 days).

The 10-day-old seedlings were moved to a plastic basin filled with sand and kept in the growth chamber at a 12/12 h (light/dark) photoperiod at 25°C ± 2°C with a light intensity of 200 μmol·m^−2^·s^−2^. Three times a week, the seedlings were watered with 1× Hoagland’s solution. After 15 days, seedlings were divided into two groups: a control group and an experimental group. The control group was watered with Hoagland’s solution every day, and the experimental group was watered with Hoagland’s solution amended with 300 mM NaCl (approximately 31.5 ms·cm^−1^) every day. To avoid the accumulation of salt in the sand media, the pot was manually irrigated daily until free drainage occurred. Determination of indexes was performed when there were significant differences between different genotypes under salt stress (13 days). For the determination of further physiological parameters and gene expression analysis, smooth bromegrass leaves were harvested, immediately frozen in liquid nitrogen, and stored at −80°C.

### Measurement of evaluation indicators and comprehensive evaluation of accessions

The number of seeds was counted to measure germination rate and germination vigor. Radicle length and embryo length were measured using a ruler. Root bud ratio, germination index, and vitality index were determined through a calculation method. Plant height was measured using a ruler. Fresh weight and biomass were measured using an electronic balance. The leaf morphology indexes were measured using the Li-3100C instrument. The root morphology indexes were measured using Epson Perfection V12000 Photo. To eliminate genetic differences, the salt tolerance coefficient was calculated based on measured indicators, and then correlation and principal component analysis were used to perform a comprehensive evaluation using the membership function.

### Measurement of physiological indicators

On the basis of the phenotype of smooth bromegrass plants, each treatment group was photographed on days 1, 5, 9, and 13 of salt treatment. Relative water content (RWC) was measured according to Ahmad et al. (2019). Chlorophyll content (Soil Plant Analysis Development (SPAD)) was measured using the SPAD-502 chlorophyll meter (China) (Li et al., 2022). Relative conductivity (REL) was measured following the method of a previous study (Wu et al., 2017). The parameters of malondialdehyde (MDA), proline (Pro), soluble protein, and soluble sugar were determined using kits from Solarbio (Beijing, China). Three biological replicates were performed.

### RNA extraction and sequencing

An RNAprep Pure Plant Kit (Tiangen, Beijing, China) was used to extract total RNA. The quality of the RNA was determined using a NanoPhotometer spectrophotometer (IMPLEN, CA, USA), Qubit 2.0 Fluorometer (Life Technologies, Carlsbad, CA, USA), and an Agilent Bioanalyzer 2100 system (Agilent Technologies, Santa Clara, CA, USA). AMPure XP Beads were used to screen the cDNA (~200 bp). After amplifying and purifying, the cDNA libraries were obtained and sequenced using the Illumina HiSeq™ 2000 System (Illumina, San Diego, CA, USA) of Metware Biotechnology Co., Ltd. (Wuhan, China). Three biological replicates were performed to sequence transcription.

### *De novo* assembly and functional annotation

The raw data were filtered using FASTP (v 0.23.2). Reads containing adapter sequences were eliminated. Paired reads were discarded if the percentage of N bases in either of the sequencing reads exceeded 10% of the total bases. Additionally, paired reads were also removed if the number of low-quality bases (Q ≤ 20) in either of the sequencing reads exceeded 50% of the total bases. Transcriptome assembly was performed using Trinity (v 2.13.2). The TransDecoder (v 5.3.0) was employed to predict coding sequence (CDS) from the transcripts assembled using Trinity. The following database was used for functional annotation of assembled unigenes: non-redundant protein sequences (Nr), non-redundant nucleotide sequences (Nt), protein family (Pfam), eukaryotic Ortholog Groups/Clusters of Orthologous Groups of proteins (KOG/COG), and a manually annotated and reviewed protein sequence database (Swiss-Prot). The annotation of the Kyoto Encyclopedia of Genes and Genomes (KEGG) pathway analysis was performed using the KEGG Automatic Annotation Server (KAAS). The Gene Ontology (GO) annotation of unigenes was performed using clusterProfiler (v 4.6.0) according to the Nr and Pfam annotation results.

### Differential unigene expression analysis

RSEM (v 1.3.1) was used to estimate gene expression levels. Based on the gene length, the fragments per kilobase of transcripts per million (FPKM) value of each gene were computed. Comparative analysis was performed according to DESeq2 v 1.22.2. The criteria for screening differentially expressed genes (DEGs) were |log_2_(Fold Change)| ≥ 2 and *p*-value < 0.01. The GO and KEGG enrichment analyses were performed based on a hypergeometric test. TF analysis was performed using iTAK (1.7a).

### Quantitative real-time PCR analyses

Nine DEGs were randomly selected for qRT-PCR analysis to verify the accuracy of the sequencing results. TRIzol Total RNA Extraction Kit (Sangon Biotech, Shanghai, China) was used to extract RNA. RNA was reverse-transcribed using an Evo M-MLV RT Mix Kit with gDNA Clean (Accurate Biotechnology, Hunan, China). The primers are shown in **Supplementary Table 2**. qRT-PCR was performed using a BioEasy Master Mix (SYBR Green) Kit (Bioer, Hangzhou, China) and a C1000 TouchChihermal Cycler system (Bio-Rad, Hercules, CA, USA). The reference gene *Actin* was used to normalize all transcripts tested. Relative transcript levels were calculated using the 2^−ΔΔCt^ method with three biological replications (Livak and Schmittgen, 2001).

### Statistical analysis

The data were sorted using Excel 2010 (Microsoft Inc., Redmond, WA, USA). The data were analyzed by one-way ANOVA using Origin 2023 (Electronic Arts Inc., San Francisco, CA, USA), followed by Tukey’s significant difference test (*p* < 0.01 or *p* < 0.05). *t*-Test was performed using SPSS 22 (SPSS Inc., Chicago, IL, USA). The figures were made using Origin 2023 (Electronic Arts Inc., San Francisco, CA, USA).

## Results

### Salinity effects on 57 smooth bromegrass growth traits

After treatment with NaCl, seedlings of the 57 smooth bromegrass genotypes were significantly suppressed (**Supplementary Table 3**). In the germination stage, the results showed that salt stress significantly reduced germination potential, germination rate, radicle length, embryo length, root bud ratio, germination index, and vitality index compared with control (**Supplementary Table 3**). In the seedling stage, NaCl significantly decreased plant height, leaf length, leaf width, leaf area, fresh weight above ground, fresh weight underground, dry weight above ground, dry weight underground, root-to-shoot ratio, root length, root project area, root surface area, average diameter, root volume, and root tips (**Supplementary Table 3**). Under the treatment of salt, there were significant differences in the growth indexes of 57 smooth bromegrass accessions, and the index values showed obvious normal distribution characteristics. Under salt treatment, the growth traits of 57 varieties were centered in a relatively small range of phenotypic values (**Supplementary Figure 1**).

### Correlation analysis of growth traits among smooth bromegrass germplasm resources under salinity

Relative values of growth characteristics of smooth bromegrass under salinity were calculated using Pearson’s correlation coefficient. These results displayed that, during the germination and seedling stages, there were 47 positive correlations (*p* < 0.01) and six negative correlations (*p* < 0.01) in the comparative values of all characteristics under salt stress (**Supplementary Figure 2**). In the germination stage, the root bud ratio did not correlate with other traits except for embryo length, showing that the root bud ratio was not a good evaluation index for smooth bromegrass. During the seedling stage, there was no significant positive correlation between average root diameter and other traits except for root volume, but a significant negative correlation with root tip and root length. From the results above, it was suggested that 20 traits could be used for the principal component analysis (PCA).

### Principal component analysis of growth traits of 57 smooth bromegrass accessions under salinity conditions

The evaluation of 57 smooth bromegrass accessions was performed by principal component analysis of the indexes among the bud stage and seedling stage. Based on the extraction method using eigenvalues greater than 1, six principal components were extracted, with the cumulative contribution rate as high as 84.515% (**Supplementary Table 4**). The screen plot clearly indicated that the first six factors were significantly higher eigenvalues (**Supplementary Figure 3**). By extracting these six factors, indicators with similar effects were grouped together, transforming the original indicators into new mutually independent composite indicators for fuzzy membership function analysis. By analyzing the principal component loading matrix, the contribution of each indicator to the composition of the principal components was observed (**Supplementary Table 5**). In the first principal component, the variables with the highest absolute loading values were root surface area and root projection area. In the second principal component, the variables with the highest absolute loading values were the germination index and germination rate. In the third principal component, the variables with the highest absolute loading values were leaf area and leaf width. In the fourth principal component, the variables with the highest absolute loading values were embryo root length and root length. In the fifth principal component, the variables with the highest absolute loading values were embryo length and radicle length. In the sixth principal component, the variables with the highest absolute loading values were radicle length and aboveground biomass.

As shown in **Supplementary Figure 4**, there was a high concentration of indicators in the germination and seedling stages. Combined with the component loading values in **Supplementary Table 5**, the important roles of the germination stage and seedling stage indicators in salt tolerance principal component analysis of smooth bromegrass were indicated. There may be a certain correlation between salt tolerances in these two stages. The salt tolerance of different smooth bromegrass accessions can be reflected more comprehensively by considering the indexes of the germination stage and seedling stage.

### Evaluation of salt tolerance among 57 smooth bromegrass germplasms

**Figure 1:**
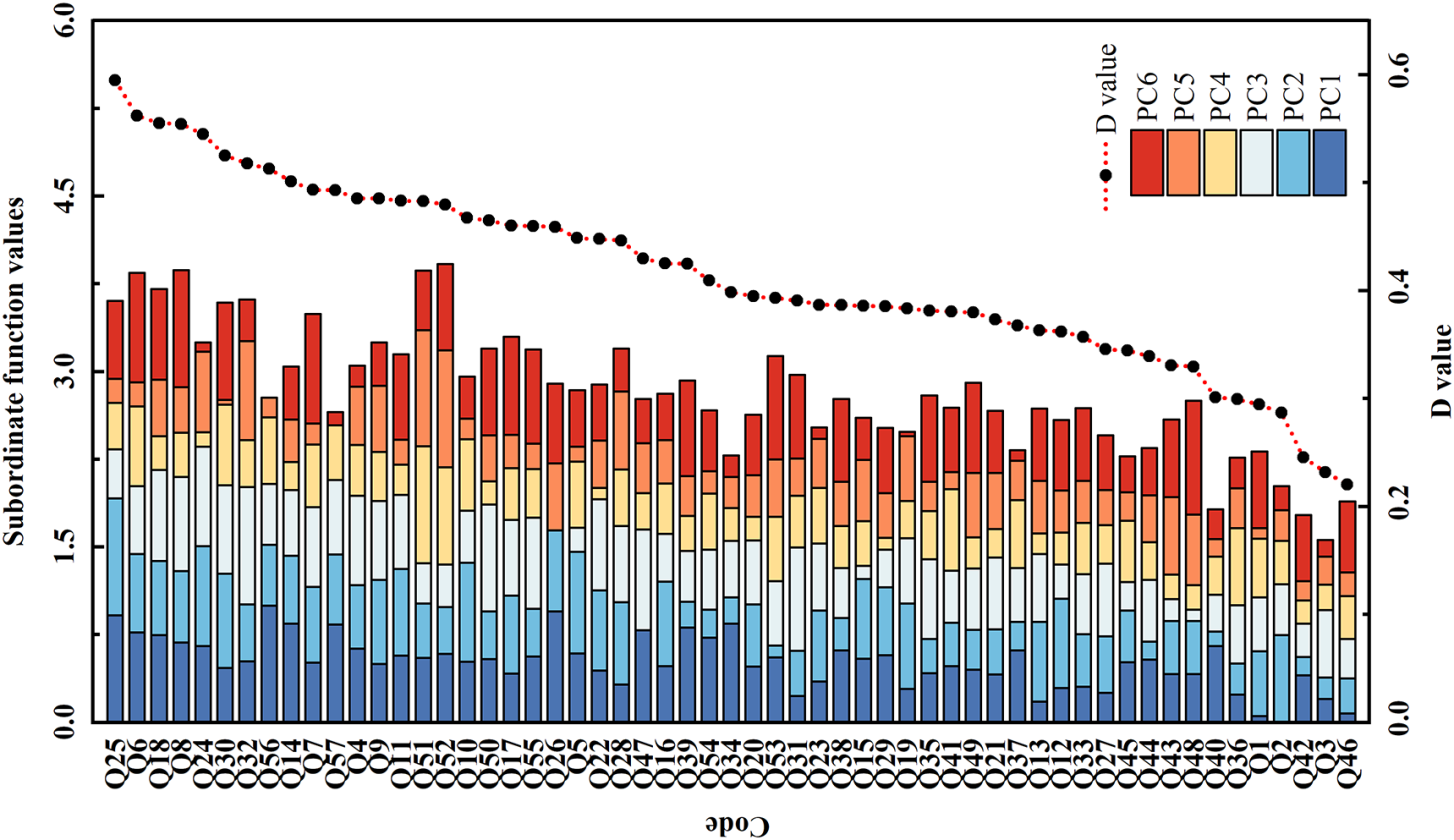
The subordinate function values analysis for comprehensive evaluation of salt tolerance among 57 smooth bromegrass accessions. Subordinate function values: the value was calculated based on the switching function vector analysis. D value: the value of weighted membership function; the higher the D value, the stronger the salt tolerance of the genotype.

In this study, the salt tolerance of 57 accessions was ranked based on the Switching Function Vector Analysis (SFVA) and D values (**Figure 1**). As can be seen in **Figure 1**, the top five genotypes with a higher salt tolerance than other accessions in the sequences were Q25, Q6, Q18, Q8, and Q24; less tolerant to salt than the other varieties were Q46 (least) and Q3 (last but one).

### Physiological analysis of smooth bromegrass seedlings under salt stress

**Figure 2:**
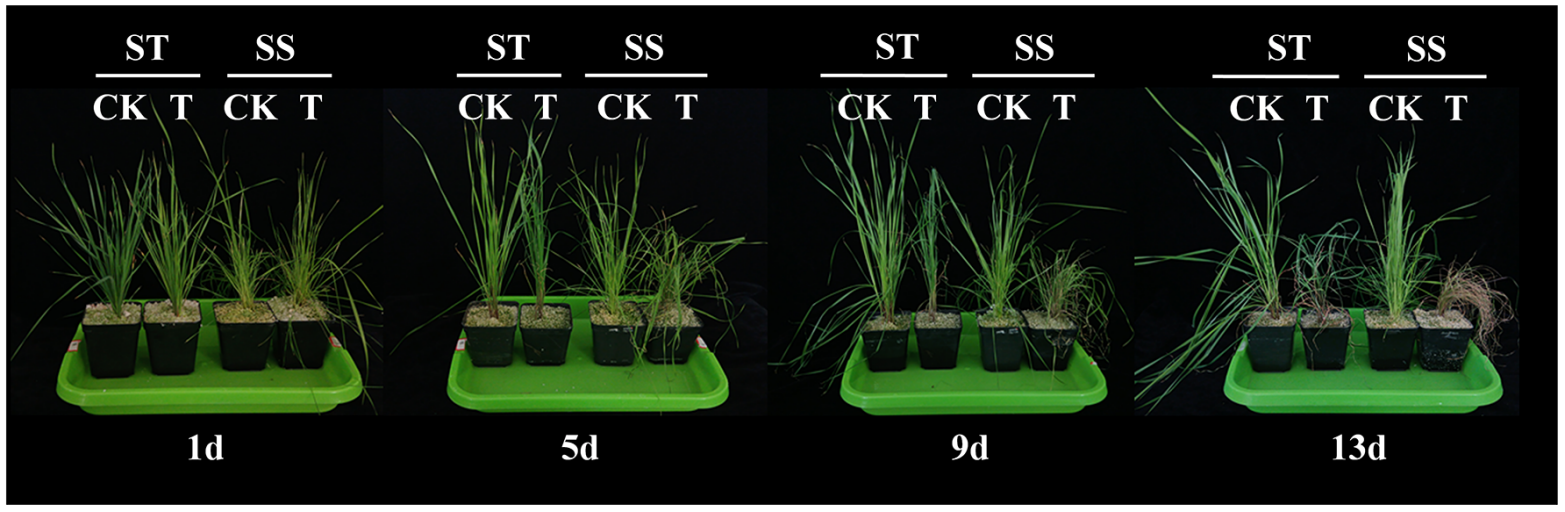
Effects of 300 mM NaCl on phenotype of smooth bromegrass seedlings under different salt stress times. ST, salt-tolerant genotype; SS, salt-sensitive genotype; CK, control treatment; T, salt treatment.

**Figure 3:**
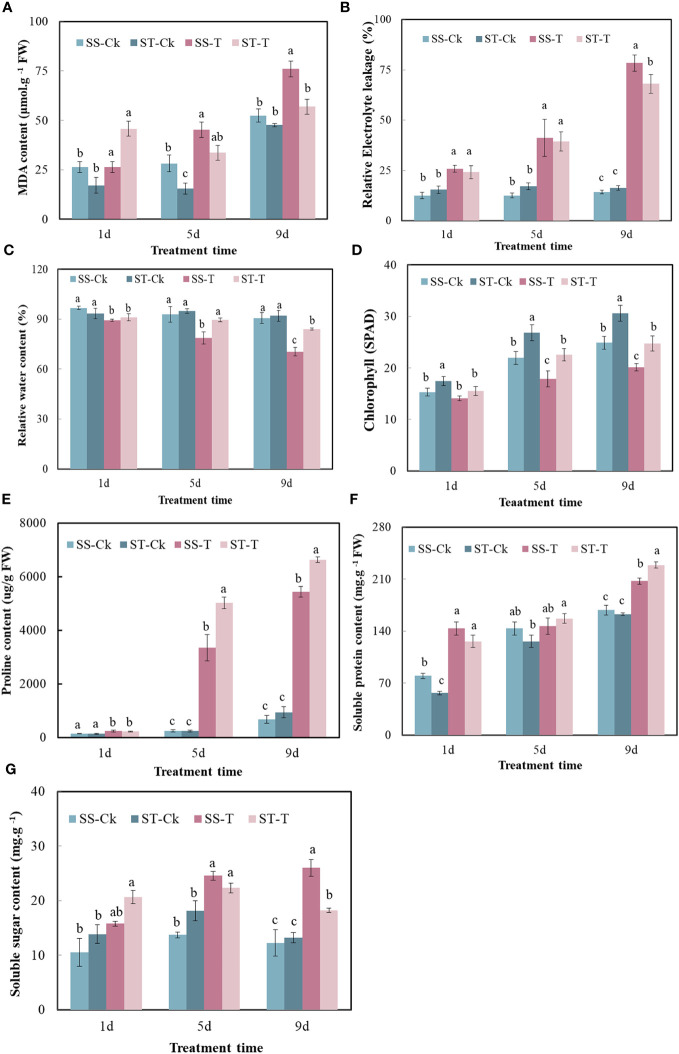
Effects of stress on physiological indexes of smooth bromegrass seedlings under different salt stress times. **(A)** MDA content. **(B)** Relative electrolyte leakage. **(C)** Relative water content. **(D)** Chlorophyll content (SPAD). **(E)** Proline content. **(F)** Soluble protein content. **(G)** Soluble sugar content. Significantly different means are shown with different letters, calculated using Tukey’s test (*p* < 0.05). ST, salt-tolerant genotype; SS, salt-sensitive genotype; CK, control treatment; T, salt treatment; MDA, malondialdehyde; SPAD, Soil Plant Analysis Development.

After 9 days of exposure to salt stress, although the leaves of the two genotypes showed different degrees of wilting, the level of wilting in the salt-tolerant genotype (ST) was lower than that in the salt-sensitive variety (SS). After 13 days of exposure to salt stress, the leaves of the SS experienced withering and eventual death (**Figure 2**). On the first day, the physiological indexes did not show any significant difference between the SS and the ST under the salt treatment. On the fifth day, there was no significant difference between SS and ST except for RWC, SPAD, and Pro under salt stress. Salt stress significantly decreased the RWC and SPAD of smooth bromegrass on the ninth day compared to the control. Under salt stress, ST had a lower content of the relative conductivity and MDA and higher contents of RWC and SPAD than SS (**Figures 3A–D**). Thus, after 9 days of salt treatment, ST showed better growth. Furthermore, in response to salt stress, salt treatment resulted in a significant increase in proline, soluble protein, and soluble sugar contents. The ST exhibited significantly higher proline and soluble protein contents compared to the SS (**Figures 3E–G**). The result showed that a key point in the level of salt treatment was reflected by 9 days of the smooth bromegrass seedlings. Hence, we selected the smooth bromegrass leaves treated with SS and ST under control and salt treatments at 9 days (serious salt stress) for transcriptome analysis.

### Transcriptome sequencing and assembly

Twelve cDNA libraries of two different genotypes of smooth bromegrass were sequenced. The clean reads of each library were obtained. The raw reads, clean reads, clean base, error rate, Q20, Q30, and GC content are shown in **Supplementary Table S6**. A total of 91.15 Gb of clean data was obtained. Each sample was 6 Gb of clean data, with a Q30 base percentage of 93% or above. A total of 360,627 transcripts with a mean length of 932 bp, an N50 of 1,446 bp, and an N90 of 392 bp, and 194,490 unigenes with a mean length of 1,197 bp, an N50 of 1,667 bp, and an N90 of 561 bp from the 12 sequenced libraries were obtained (**Supplementary Table 7**). Transcripts and genes were grouped by length as shown in **Supplementary Figure 5**.

### Analysis of gene function annotation

To explore the functionality of unigenes, the sequences of all obtained unigenes were compared using databases such as Nr, Pfam, KOG, SwissProt, KO, NT, and GO, and annotation information for all unigenes was obtained. Annotated unigenes numbers and percentages of the total are listed in **Supplementary Table S8**. A total of 138,481 assembled unigenes, 71.2% of the total, were annotated in at least one databank. It was found that all unigenes showed the highest homology with *Triticum aestivum* by the analysis of 134,652 unigenes annotated in the Nr database, indicating a close relationship with *T. aestivum* (**Supplementary Figure 6**).

### Expression and cluster analysis of the unigenes

**Figure 4:**
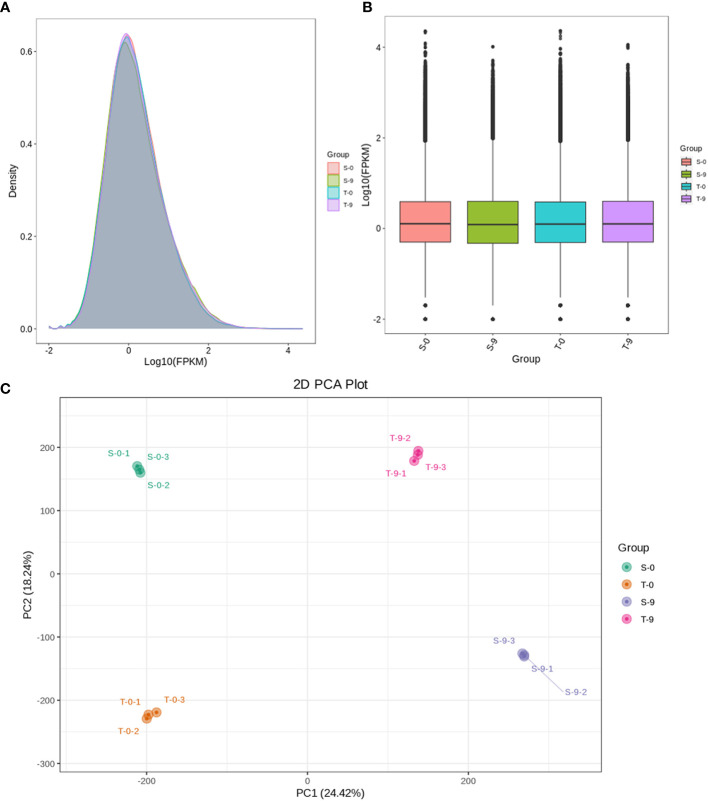
Comparison of DEG expression levels of different treatments and PCA with different treatments in smooth bromegrass. **(A)** Density map: the horizontal axis is the log10 (FPKM) value of the gene, and the vertical axis is the distribution density of the gene corresponding to the expression amount. **(B)** FPKM distribution: the middle horizontal line of the box is the median, the upper and lower edges of the box are 75%, and the upper and lower limits are 90%. The external shape is the kernel density estimation. **(C)** PCA. DEG, differentially expressed gene; PCA, principal component analysis.

The samples were divided into four groups—salt-sensitive and salt-tolerant samples under control (S-0 and T-0) and salt-sensitive and salt-tolerant samples under stress (S-9 and T-9)—to analyze the transcriptome differences. To analyze the expression level of genes, the read counts of the smooth bromegrass transcriptome were converted to FPKM. Some of the gene expression levels in the T-9 were higher than in S-9 (**Figures 4A, B**). The gene expression density diagram of samples at different genotypes after salt exposure indicated a similar trend in gene abundance and gene expression density. Moreover, the log_2_FPKM values were concentrated in the [−2, 2] interval for all transcripts of the samples (**Figure 4A**). Correlation heat map analysis of gene expression levels between samples showed a high degree of agreement between each group of samples to ensure the reliability of subsequent differential gene analysis (**Supplementary Figure 7**). PCA showed that the significant difference between salt and control stress was mainly caused by PC1 (24.42%), while the difference between genotypes was mainly caused by PC2 (18.24%) (**Figure 4C**).

### Differential expression analysis of genes

**Figure 5:**
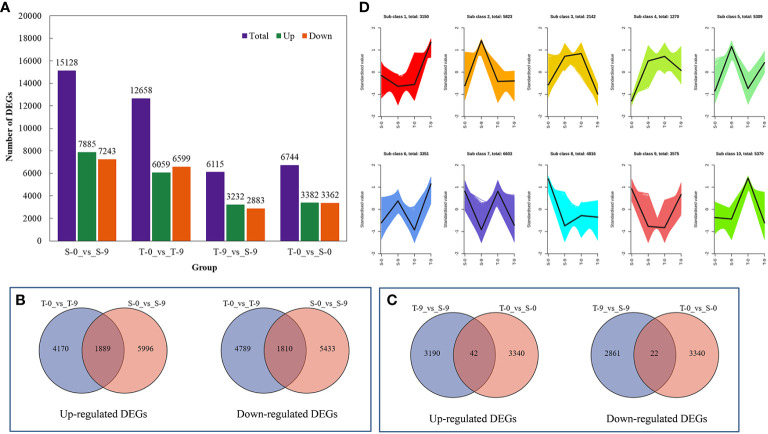
The comprehensive description of the transcriptome analysis of the salt stress response of smooth bromegrass. **(A)** Bar charts of up- and downregulated genes in groups. **(B)** Venn diagram of upregulated and downregulated DEGs in the comparison group of T-0_vs_T-9 and S-0_vs_S-9. **(C)** Venn diagram of upregulated and downregulated DEGs in the comparison group of T-9_vs_S-9 and T-0_vs_S-0. **(D)** K-mean cluster analysis. DEGs, differentially expressed genes.

The four groups (S-0_vs_S-9, T-0_vs_S-0, T-0_vs_T-9, and T-9_vs_S-9) had 15,128, 6,744, 12,658, and 6,115 DEGs, respectively; 7,885, 3,382, 6,059, and 3,232 genes, respectively, were upregulated, and 7,243, 3,362, 6,599, and 2,883 genes, respectively, were downregulated (**Figure 5A** and **Supplementary Table 9**). This displayed that, under salt stress, the gene expression levels were significantly changed between salt-tolerant and salt-sensitive varieties. In T-9 and S-9, 1,889 common unigenes were upregulated and 1,810 were downregulated in the Venn diagram analysis (**Figure 5B**). T-9 had 4,170 unique upregulated DEGs, and S-9 had 5,996 unique downregulated DEGs. These upregulated DEGs, unique to T-9, may have a role in salinity resistance, while the upregulation of DEGs may have a correlation with salt stress susceptibility in S-9. For the two comparisons of T-9_vs_S-9 and T-0_vs_S-0, 3,190 unique genes were specifically upregulated in T-9 and may contribute to salt stress resistance (**Figure 5C**). In the current study, thousands of DEGs were discovered, which can be attributed to the complex genetic background. All DEGs were divided into 10 subclasses by K-means clustering analysis, among which subclass 7 was the largest, containing 6,603 DEGs, and subclass 4 was the smallest, containing 1,270 DEGs (**Figure 5D**).

### Transcription factor analysis

**Figure 6:**
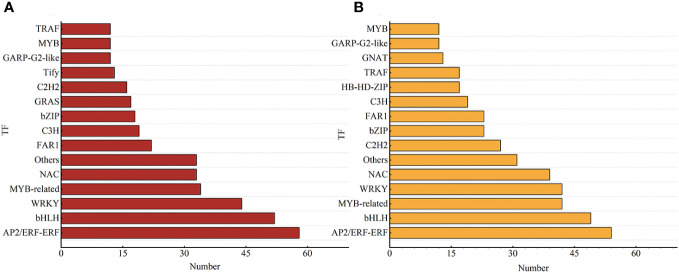
Different TFs of smooth bromegrass in response to salt stress. **(A)** T-0_vs_T-9. **(B)** S-0_vs_S-9. The chart shows the different kinds of transcription factors (TFs) on the vertical axis and the quantity of transcription factors on the horizontal axis.

Further investigation was conducted on the transcripts of TF-encoding genes to explore the regulatory mechanisms of salt stress on smooth bromegrass. A total of 567 TF-encoding genes from 59 different families in T-0_vs_T-9 were identified. As shown in **Figure 6** and **Supplementary Table 10**, the top four DEGs in numerical order were 58 in the AP2/ERF-ERF family, 52 in the bHLH family, 44 in the WRKY family, and 34 in MYB-related families. A total of 622 TF-encoding genes were identified from 62 different families in S-0_vs_S-9. The top four were arranged in ascending order of quantity: 54 in the AP2/ERF-ERF family, 49 in the bHLH family, 42 in the MYB-related family, and 42 in the WRKY family. In two varieties, the most represented TFs were AP2/ERF-ERF, and a total of 112 AP2/ERF-ERF-related genes were enriched in these two genotypes.

### Functional annotation and enrichment analysis of DEGs

**Figure 7:**
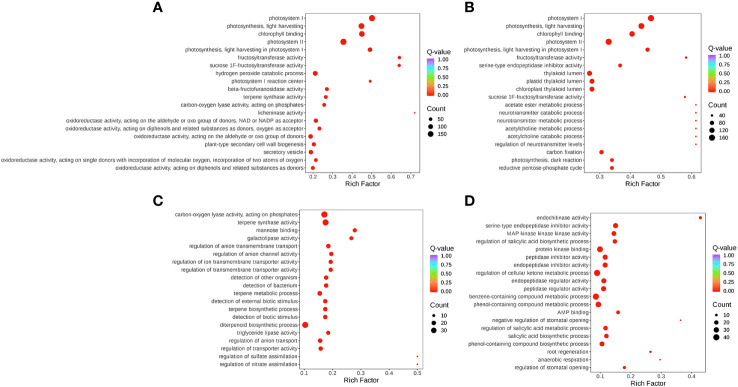
GO pathway enrichment plot for gene expression during salt stress. **(A)** T-0_vs_T-9. **(B)** S-0_vs_S-9. **(C)** S-0_vs_T-0. **(D)** S-9_vs_T-9. The size and color of the circle indicate the number of transcripts and the significance value (*p*-value) of the rich factor, respectively. GO, Gene Ontology.

**Figure 8:**
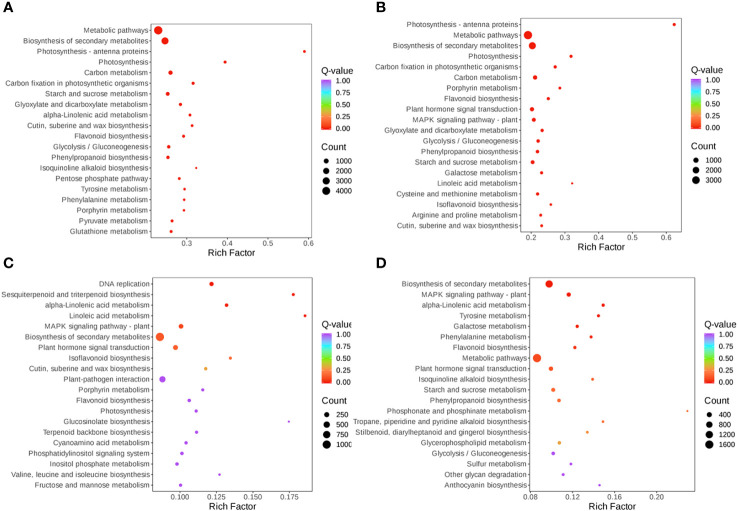
Scatterplot of KEGG pathway enrichment for DEGs under salt stress. **(A)** S-0_vs_S-9. **(B)** T-0_vs_T-9. **(C)** S-0_vs_T-0. **(D)** S-9_vs_T-9. The size and color of the circle indicate the number of transcripts and the significance value (*p*-value) of the rich factor, respectively. KEGG, Kyoto Encyclopedia of Genes and Genomes; DEG, differentially expressed gene.

The expression of DEGs in different samples was identified by comparative analysis and enrichment analysis. Using bioinformatics databases such as the GO and KEGG can acquire the most relevant biological pathways and DEGs. In the GO database, DEGs in S-0_vs_S-9 and T-0_vs_T-9 were significantly enriched in “fructosyltransferase activity”, “sucrose 1F-fructosyltransferase activity”, and “photosystem I” (**Figure 7** and **Supplementary Table 11**). In the comparisons of S-9_vs_T-9, “endochitinase activity” was highly enriched (**Figure 7** and **Supplementary Table 11**). In the KEGG database, enrichment analysis of DEGs indicated that there were 14 common pathways between S-0_vs_S-9 and T-0_vs_T-9, among which “Photosynthesis-antenna proteins” and “Photosynthesis” were the most enriched (**Figure 8** and **Supplementary Table 12**). In addition, in the comparisons of S-9_vs_T-9, the phosphonate and phosphinate pathways were highly enriched. Compared with S-0_vs_S-9, six significant enrichment pathways were uniquely found in T-0_vs_T-9: “plant hormone signal transduction”, “MAPK signaling pathway—plant”, “Galactose metabolism”, “Linoleic acid metabolism”, “Cysteine and methionine metabolism”, and “Arginine and proline metabolism”. The profile of DEGs enriched in the KEGG pathways compared to S-0_vs_T-0 was similar to S-9_vs_T-9. Two common enriched pathways, “plant hormone signal transduction” and “MAPK signaling pathway—plant”, were obtained among the three comparisons of T-0_vs_T-9, S-0_vs_T-0, and S-9_vs_T-9.

### Analysis of DEGs associated with plant hormone signal transduction and MAPK signaling pathway—plant pathways

In order to ensure the reliability and representativeness of the selected DEGs, the filtering standard was defined as *p*-value < 0.01 and |log_2_(Fold Change)| ≥ 4. Then, two enriched KEGG pathways, “plant hormone signal transduction” and “MAPK signaling pathway—plant”, were further compared.

**Figure 9:**
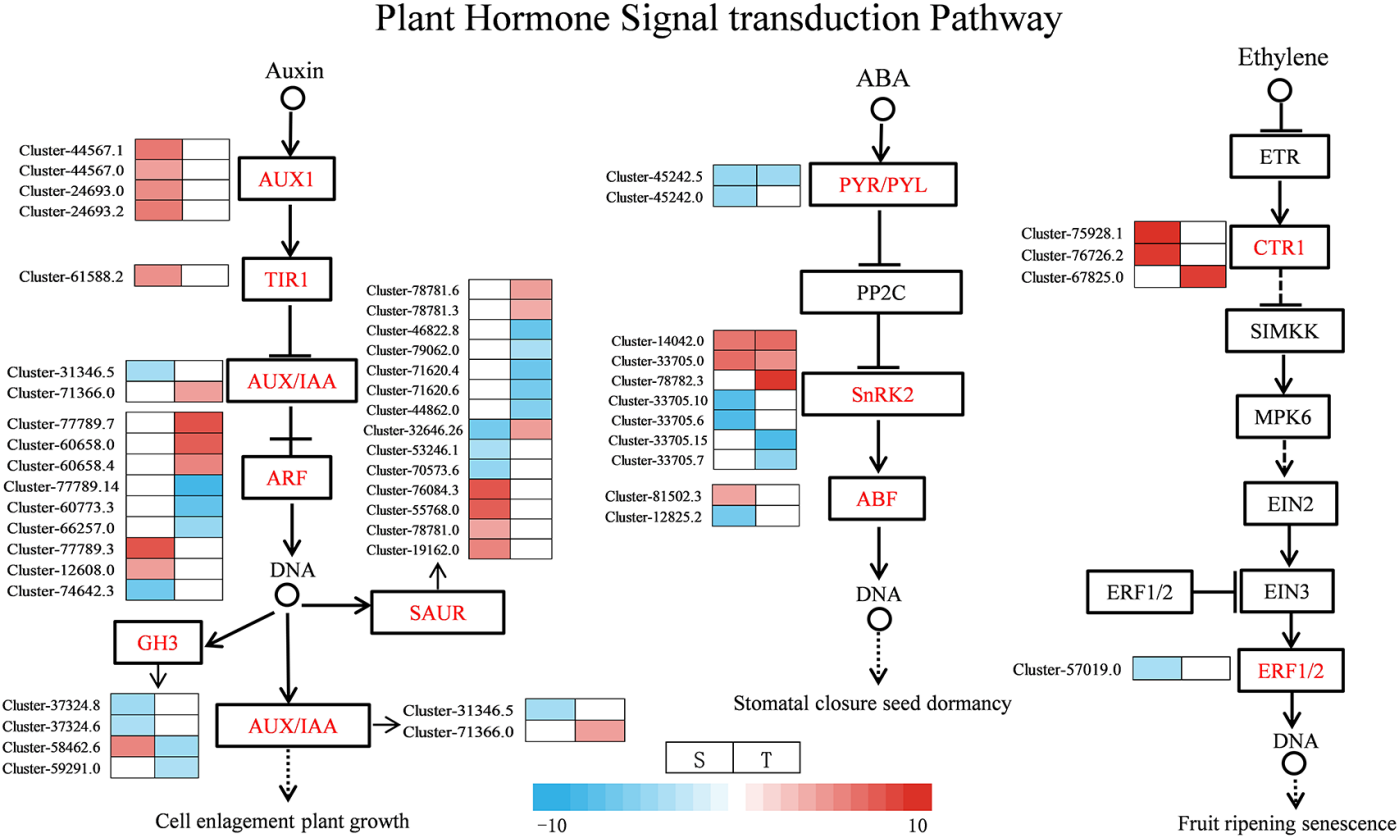
Expression map of genes related to plant hormone signaling transduction. S, salt-sensitive genotype; T, salt-tolerant genotype.

**Figure 10:**
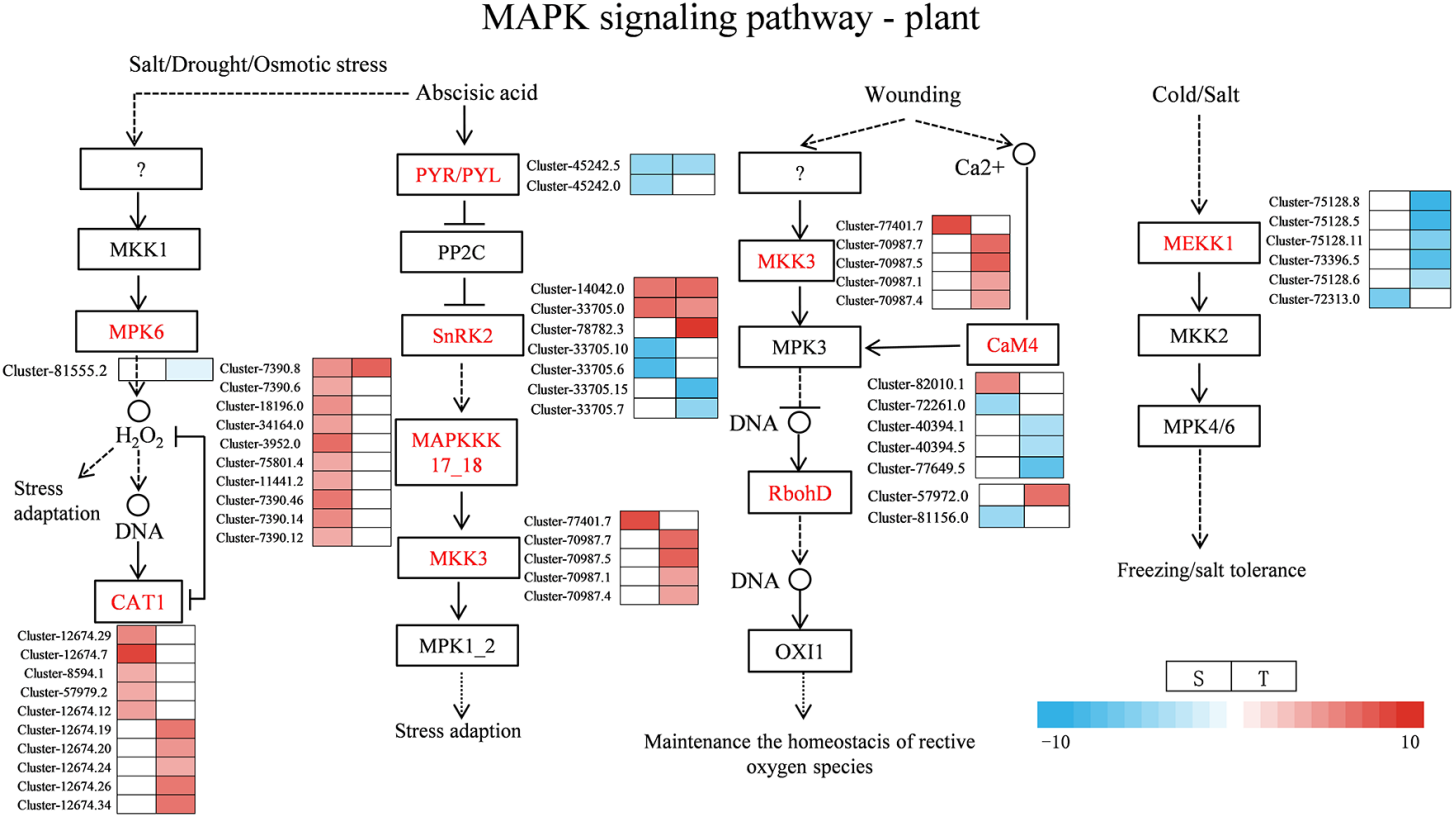
Heat map of gene expression related to MAPK signal pathway—plant. S, salt-sensitive genotype; T, salt-tolerant genotype.

Under salt stress, several DEGs related to plant hormone signal transduction were found to be enriched in the two genotypes, such as ABA, IAA, and ETH. Most of the DEGs were involved in ABA, auxin, and ETH signaling pathways as shown in **Figure 9** and **Supplementary Table 13**. For the ABA signal, two SNF-related protein kinase 2 (*SnRK2*) were upregulated in the salt-sensitive variety, three genes were upregulated in the salt-tolerance variety, and one *ABF* gene was upregulated and one was downregulated in the salt-sensitive variety. Moreover, two *PYR/PYL* genes were downregulated in the salt-sensitive variety, and one was downregulated in the salt-tolerant variety. For the auxin signal, the four DEGs encoding AUX1 and one encoding TIR1 were upregulated in salt-sensitive variety. One DEG encoding AUX/IAA was downregulated in the salt-sensitive variety and upregulated in the salt-tolerant variety. In addition, six genes encoding auxin response factor (*ARF*) were induced in the salt-tolerance genotype but three in the salt-sensitive variety. For the ETH signal, serine/threonine-protein kinase (*CTR1*) was upregulated in two genotypes, whereas the ethylene-responsive transcription factor 1B (*ERF1B*) was downregulated in the salt-sensitive variety under salt stress. Disease resistance and ROS scavenging have been linked to the MAPK pathway. Two DEGs were downregulated and two DEGs were upregulated encoding SnRK2 in the salt-sensitive variety, whereas two DEGs were downregulated and three DEGs were upregulated encoding SnRK2 in the salt-tolerant variety (**Figure 10** and **Supplementary Table 14**). A respiratory burst oxidase gene (*Rboh D*) is downregulated in the salt-sensitive variety, and another was upregulated in the salt-tolerance variety. Five CAT1-related genes were all upregulated in salt-sensitive and salt-tolerance accessions. Interestingly, five MEKK1-related genes were downregulated to enhance salt tolerance.

### Analysis of DEGs associated with photosynthesis and other specific interest pathways

**Figure 11:**
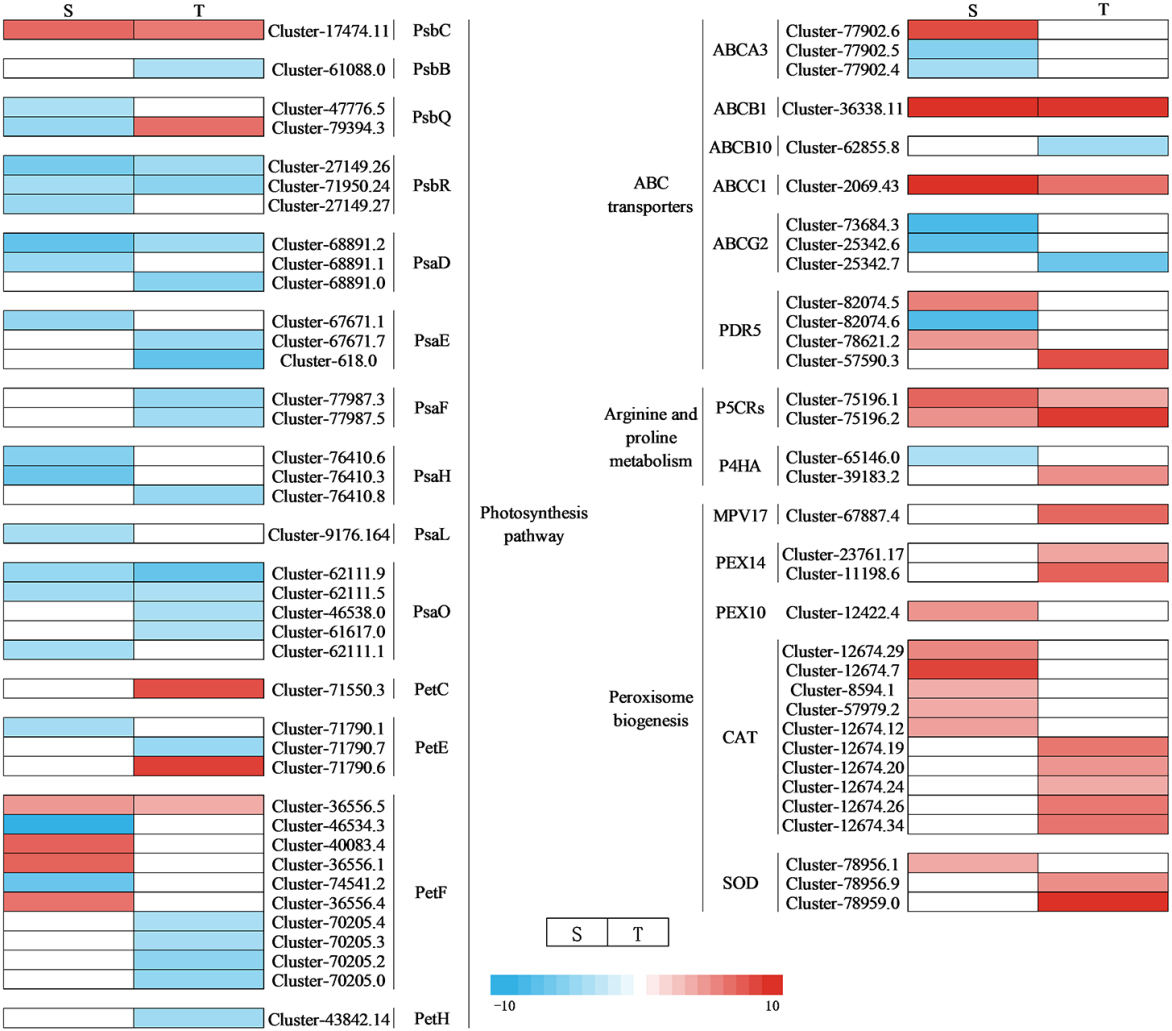
Heat map of DEG expression associated with photosynthetic, antioxidant, ROS, and ABC transport pathways under NaCl stress. S, salt-sensitive genotype; T, salt-tolerant genotype; DEG, differentially expressed gene; ROS, reactive oxygen species.

Through DEG annotation analysis, 39 genes that encode photosynthesis-related proteins were identified (**Figure 11** and **Supplementary Table 15**). There were 14 DEGs involving the photosystem II protein of differential varieties that were downregulated under salt stress, while one gene was upregulated in a salt-sensitive genotype, and two were in another. Five genes encoding photosynthetic electron transport were upregulated in the salt-sensitive variety, while two genes were upregulated in the salt-tolerant genotype. In addition, one gene encoding cytochrome b6-f complex iron–sulfur subunit protein (Pet C) was upregulated in the salt-tolerant genotype. In addition, the following were identified: DEGs associated with antioxidant enzymes (*SOD*, *CAT*, *PEX10*, *PEX14*, and *MPV17*), proline synthase (*P5CRs* and *P4HA*), and ABC transporters (*ABCA3*, *ABCB1*, *ABCB10*, *ABCC1*, *ABCG2*, and *PDR5*). They were all regulated with different levels under NaCl stress (**Figure 11** and **Supplementary Table 16**). In our research, there were 10 genes encoding these three types of zinc finger proteins, five of which were upregulated in the salt-tolerant genotype. One gene encoding glutathione was upregulated in the salt-tolerant genotype. Twenty glutathione *S*-transferase, N-terminal domain-related genes were identified to be regulated under salt stress. Three pyridine nucleotide-disulfide oxidoreductase-, one calcium-dependent channel-, one voltage-gated chloride channel-, and two DELLA protein-related genes were identified under salt stress, indicating the key genes responsible for salt tolerance in two genotypes.

### RNA-seq data validation with qRT-PCR

The trend of qRT-PCR expression was consistent with the trend of RNA-seq (FPKM) (**Supplementary Figure 8A**). Meanwhile, qRT-PCR data displayed a strong correlation with the RNA-seq in both SS (R^2 = ^0.9185) and ST (R^2 = ^0.9511) (**Supplementary Figure 8B, C**), confirming the high accuracy of the RNA-seq expression pattern. These results confirmed the reproducibility and reliability of RNA-seq data.

## Discussion

Salinity can inhibit plant growth, disrupt cell structure, increase mortality rates, and prevent the completion of the life cycle (Sharifi, 2010; Zhang and Shi, 2013). Yin et al. (2022) found that salt stress affected the growth of bermudagrass by increasing relative electrolytic leakage, decreasing chlorophyll content, and increasing Na^+^ accumulation. Similarly, the shoot and root growth of seven genotypes of zoysiagrass (*Zoysia* spp.) with different tolerance to salt stress were reduced, and the leaf senescence was accelerated (Hooks et al., 2022). In our research, the growth and development of smooth bromegrass seedlings was retarded due to the reduction of RWC and SPAD and the accretion of MDA content. Meanwhile, compared to the salt-sensitive variety, our results also found that the relative water content and chlorophyll SPAD content of the salt-tolerant variety were higher, the accumulation of relative conductivity and MDA content were lower, and the contents of soluble protein and proline were higher when exposed to salt stress (**Figures 3A–F**). These results suggested that the salt-tolerant genotype possessed stronger osmotic regulation and photosynthetic regulation ability to cope with adversity.

It is difficult for a single trait to reflect the salt tolerance of a plant, as it is a comprehensive, quantitative, and complex trait influenced by multiple environmental and genetic factors (Al-Ashkar et al., 2020). However, PCA and membership function methods have generally been regarded as relatively reliable methods for evaluating salt tolerance (Baksh et al., 2021). Zhang et al. (2022) conducted a salt tolerance evaluation of 100 oat germplasm samples by measuring 10 parameters including chlorophyll content and root/shoot ratio (fresh and dray water). Furthermore, they employed PCA and membership functions to comprehensively assess and screen for salt-tolerant and salt-sensitive germplasms (Zhang et al., 2022). There are currently few studies evaluating salt tolerance combined with germinating and seedling stages. In this work, the comparison among 57 accessions suggested that there were large differences among 22 traits under salt stress (**Supplementary Table 3**). Pearson’s correlation coefficients for growing traits among 57 smooth bromegrass accessions suggested that 20 traits could be recognized as evaluation traits for salt tolerance by PCA (**Supplementary Figure 2**). Q25 and Q46 were classified as the most salt-tolerant accession and salt-sensitive accession to explore their salt response mechanisms by transcriptome profiling.

Transcriptome data from this study showed that stress up- and downregulated a great number of DEGs, resulting in the activation of DEGs to resist stress (Li et al., 2022). The salt-sensitive genotype had 2,470 DEGs more than the salt-tolerant genotype, which may be attributed to an increased demand for the salt-sensitive genotype to activate a large number of genes in response to salt-induced stress (**Figure 5A**). We also found that the photosynthesis pathway was enriched by KEGG and GO enrichment analyses (**Figures 7**, **8**). Similarly, for the salt-tolerant genotype, plants improved photosynthesis under salt stress due to the related DEGs enriched in the photosynthetic processes (**Supplementary Table 15**). This finding was consistent with a previous study showing that under salt stress transgenic rice increased the rate of photosynthesis due to the enrichment of DEGs involved in photosynthesis (Boonchai et al., 2018). In contrast, “plant hormone signal transduction” and “MAPK signaling pathway—plant” pathways may correlate with smooth bromegrass resistance to salt stress (**Figure 8**).

**Figure 12:**
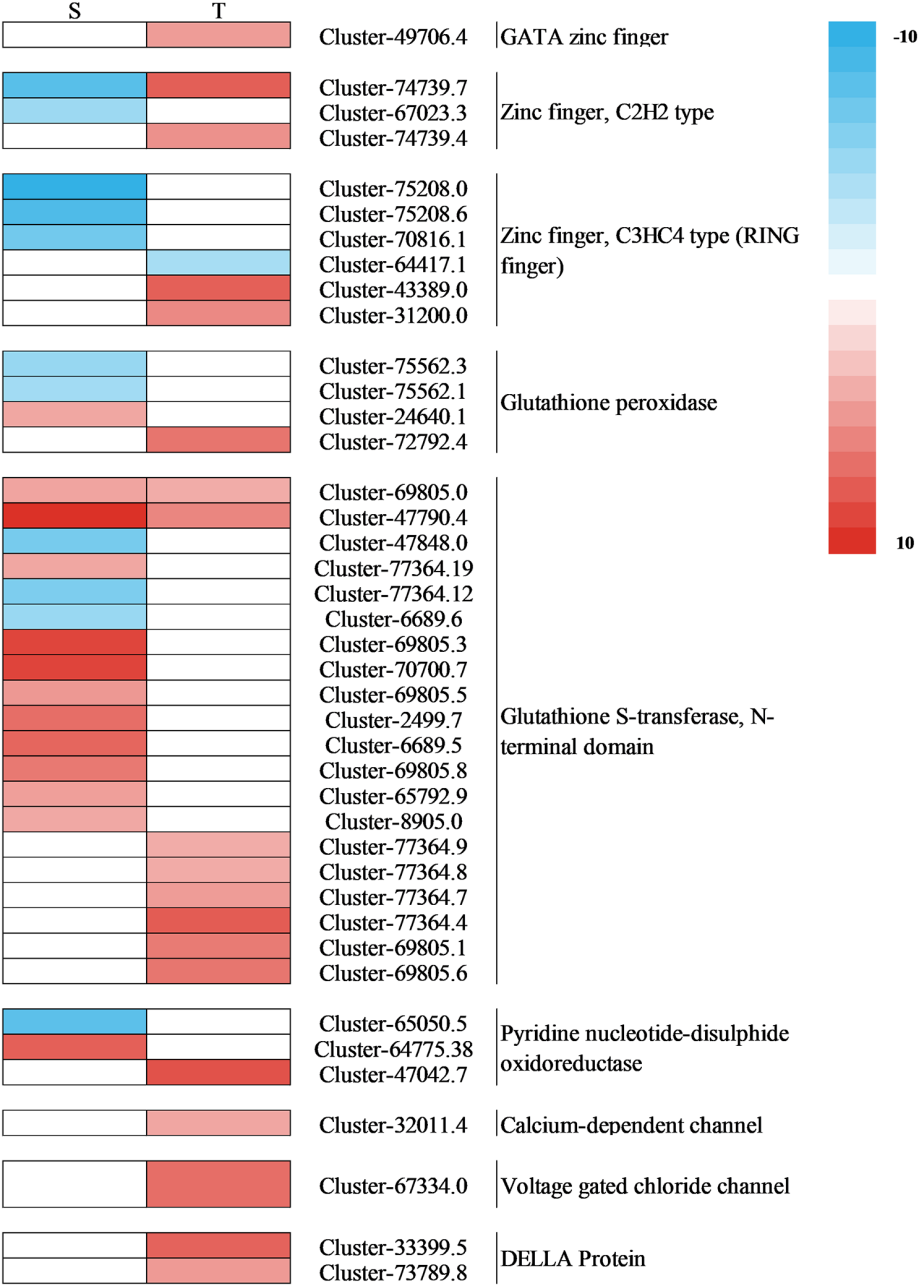
Heat map of the expression of the DEGs associated with interested pathways under salt stress. S, salt-sensitive genotype; T, salt-tolerant genotype; DEGs, differentially expressed genes.

Plant hormones, especially auxins, are important for regulating plant development in coping with salt stress (Li et al., 2022). The auxin-responsive genes can be divided into three main classes of genes: *Aux/IAA*, *GH3*, and *SAUR* (Hagen and Guilfoyle, 2002). Through the transcription of *Aux/IAA* genes, stress pathways interact with the auxin gene regulatory network (Shani et al., 2017). In this research, for the salt-tolerant variety, stress induced the expression of *Aux/IAA* and *ARF3*, which may be the reason for the salt-tolerant genotype, and had the ability to endure the salt stress. ABA is one of the key hormones in plant stress response and regulates intracellular water balance through stomatal closure (Morgil et al., 2019). A previous study showed that when PYR/PYL/RCAR bind to ABA, the complex reduces the inhibitory activity of SNF1-associated kinases (SnRKs) interacting with PP2C and activating its downstream transcription factors (Yang et al., 2019). In the regulation of plant responses to osmotic stress, SnRK2s play a crucial role under stress (Di et al., 2018). The closure of the stomata of tomato is positively regulated by DELLA proteins (Sukiran et al., 2020). Among the DELLA protein, there were two gene expressions in the salt-tolerant genotype (**Figure 12**). Their effect was strengthened by ABA, which plays an important role in the mediation of resistance (Sukiran et al., 2020). In this study, the salt-tolerant genotype adapted to the stress by accumulating ABA and upregulating DELLA protein under salt stress, leading to the regulation of cells and reduction of water loss to cope with salt stress. These findings were consistent with physiological observations (**Figure 3C**). The accumulation of ethylene under salt stress plays a crucial role in salt response, and the negative regulation of *CTR1* leads to enhanced salt tolerance (Yu et al., 2020). The ERFs have been identified as the most important downstream regulators of the ETH signaling pathway in the stress response, and the *ERF1B* has been identified as a positive regulator of salt stress tolerance (Gao et al., 2015). In this study, there were two CTR1-related genes upregulated and one ERF1B-related gene downregulated. Therefore, it was inferred that the salt-tolerant genotype might enhance their salt tolerance by regulating key genes involved in ethylene regulation.

Salt stress also increased the accumulation of Na^+^ of cells in photosystem II, thereby reducing the photosynthesis of salt-sensitive chickpea (Khan et al., 2016; Kotula et al., 2019). Psb Q and Psb P play an important role in the photosystem in response to abiotic stress (Vani et al., 2001; Adams et al., 2013). In our study, one Psb Q-related gene was upregulated to cope with salt stress. Ferredoxin (Pet F), a catalytic enzyme in the electron transfer chain of the photosynthetic system, actively participates in the process of carbon assimilation (Fukuyama, 2004). In this study, genes related to Pet F were upregulated, suggesting that the sensitive genotype was subjected to damage under salt stress, and the disruption of the electron transfer system increased the production of proteins required to maintain photosynthesis. Studies have indicated that plastocyanin, a copper-containing protein, plays an important part in electron transfer. It actively participates in homeostatic regulation and exhibits antioxidant functions (Zhou et al., 2018). This study observed that the gene encoding cytochrome b6-f complex iron–sulfur subunit protein (*Pet C*) upregulated in the salt-tolerant genotype. This was in line with our finding: salt stress decreased the content of chlorophyll considerably, and the salt-tolerant genotype had significantly more chlorophyll than the salt-sensitive genotype.

MAPK balances the production of reactive oxygen species (Matsuoka et al., 2018). Under stomatal adaptation stress, the MAPK pathway was involved in the ABA signaling pathway, with ROS acting as a second messenger in this pathway (Matsuoka et al., 2018). NaCl may be responsible for inducing *Rboh D* overexpression, as a response element to release H_2_O_2_ in the respiratory burst, triggering the expression of relevant genes by eliminating H_2_O_2_ to keep intracellular homeostasis intact (Dietz et al., 2016). It has been discovered that in the fight against salt stress, GATA zinc finger proteins were closely linked to ABA signaling and ROS scavenging (Gupta et al., 2017). In our research, salt stress induced the transcription levels of genes related to Rboh D (**Figure 10**) and GATA zinc finger protein (**Figure 12**) in the salt-tolerant genotype to cope with salt stress. Physiological data suggested that stress-induced oxidative damage led to a significant decrease in chlorophyll content and RWC and elevated relative conductivity. Hence, by modulating the levels of osmoprotectants (proline, soluble protein, and sugar), smooth bromegrass activated its antioxidant defense system (**Figure 3**). The transcriptome data also revealed the molecular level activation of the antioxidant defense system in response to NaCl stress. The KEGG analysis showed enrichment of arginine and proline metabolism pathways and hormone signaling following NaCl treatment (**Figure 8**). Furthermore, the number of DEGs associated with antioxidant enzymes (*SOD* and *CAT*) and proline synthesis enzymes (*P5CRs* and *P4HA*) was also modulated under NaCl stress and induced overexpression in salt-tolerant variety (**Figure 11**). These physiological indicators were correlated with transcriptome data, strongly indicating that these related DEGs play a crucial role in response to stress and subsequent protection against ROS damage.

In plants, the negative electric forces triggered by salt stress could be controlled by sodium entering through ion channels or other membrane transport proteins that increase the movement of sodium across the plasma membrane (Ma et al., 2022). Studies found that ABC transporters play a crucial role in transporting substances across biological membranes in plants, using energy from ATP to move them in and out (Kang et al., 2010; Nguyen et al., 2014). In our research, salt stress induced the transcription levels of genes related to ABC transporters to cope with stress to decrease the passage and transfer of sodium (**Figure 11**). Meanwhile, we also discovered a gene encoding a calcium-dependent channel and a voltage-gated chloride channel (CLC) gene in the salt-tolerant genotype. The upregulation changes observed in the salt-tolerant genotype showed that these genes were involved in the salt tolerance of smooth bromegrass (**Figure 12**). Furthermore, we also identified several key genes. *ZFP179*, which encodes a C2H2-type zinc figure protein in rice, has been used to cope with salt stress (Sun et al., 2010), and *BrRZFP1*, which encodes a C3HC4-type protein in *Brassica rapa*, has been used for stress adaptation (Jung et al., 2013). Glutathione (GSH) is a crucial component that is involved in antioxidant defense under stress (Sun et al., 2023). Glutathione *S*-transferases (GSTs) are important and common enzymes that play an important role in the detoxification of cells under stress. Sharma et al. (2014) showed that *GST* overexpression significantly reduced ROS production and oxidative damage. In addition, the upregulation of pyridine nucleotide-disulfide oxidoreductase genes may display a higher expression of the glutathione reductase (GR) transcripts, resulting in improving GR activity and antioxidant capacity against salt stress (Trivedi et al., 2013; Mudalkar et al., 2017). The results obtained in this research confirmed these findings where four genes involved in these two types of zinc finger proteins, a gene involved in GSH, eight genes involved in GSTs, and a gene involved in pyridine nucleotide-disulfide oxidoreductase were identified to be regulated in salt-tolerant genotype under NaCl stress (**Figure 12**).

TFs are commonly known to be key genes that respond to stress. TFs, such as AP2/ERF, WRKY, and bHLH, regulate the expression of salt-responsive genes and ultimately determine the level of salt tolerance in the plant (Zhang et al., 2009; Liang et al., 2017). In this study, the AP2/ERF-ERF was the most represented transcription factor among the two genotypes (**Figure 6** and **Supplementary Table 10**). AP2/ERF has been shown to play an important role in the response to biotic and abiotic stresses and is one of the largest families of transcription factors in the plant genome (Zhu et al., 2023). ERFs were important regulators of abiotic stress responses, especially ethylene and salt stress (Li et al., 2020). The WRKY transcription factor is essential for stress resistance (Wang et al., 2023). Yu et al. (2023) showed that *OsWRKY53* could negatively regulate *OsMKK10.2* and *OsHKT1;5* to coordinate the regulation of saline–alkaline stress in rice. In this study, WRKY played a certain role in salt stress. Interestingly, most WRKY transcription factors in the salt-tolerant genotype were downregulated to cope with salt stress, while most were upregulated in the salt-sensitive genotype, suggesting that this might better enhance the resistance of smooth bromegrass.

## Conclusion

The salt tolerance and sensitivity of 57 smooth bromegrass genotypes were evaluated during the germination and seedling stages. Among them, Q25 exhibited the highest salt tolerance, while Q46 was identified as the most salt-sensitive variety compared to others. Under salt stress, Q25 not only maintained lower relative conductivity and MDA content (*p* < 0.05) but exhibited substantially better performance than Q46; the contents of leaf RWC, chlorophyll, and proline were also significantly higher than Q46 (*p* < 0.05). A large number of candidate genes have been identified that were associated with salt tolerance in smooth bromegrass. DEGs participated in the phytohormone signaling pathway, transcription factors, modulation of osmoprotectants, photosynthesis, and so on, contributing to salt stress adaptation in smooth bromegrass. This result has provided genetic resources and a theoretical basis for future salt tolerance breeding, as well as provided useful information for understanding the molecular regulations related to salt tolerance in smooth bromegrass.

## Data availability statement

The data presented in the study are deposited in the National Center of Biotechnology Information (NCBI) repository, accession number Bioproject PRJNA1017383.

## Author contributions

WS: Writing – original draft, Writing – review & editing. XG: Writing – review & editing. HL: Writing – review & editing. SL: Writing – review & editing. JW: Writing – original draft. XW: Writing – original draft. TW: Writing – original draft. YY: Writing – original draft. PH: Writing – original draft. XL: Writing – original draft. BF: Writing – review & editing.

## References

[B1] Adams W. W. Muller O. Cohu C. M. Demmig-Adams B. (2013). May photoinhibition be a consequence, rather than a cause, of limited plant productivity? Photosynth. Res. 117, 31–44. doi: 10.1007/s11120-013-9849-7 23695654

[B2] Ahmad S. Kamran M. Ding R. Meng X. Wang H. Ahmad I.. (2019). Exogenous melatonin confers drought stress by promoting plant growth, photosynthetic capacity and antioxidant defense system of maize seedlings. Peer J. 7, e7793. doi: 10.7717/peerj.7793 31616591 PMC6791350

[B3] Al-Ashkar I. Alderfasi A. Ben Romdhane W. Seleiman M. F. El-Said R. A. Al-Doss A. (2020). Morphological and genetic diversity within salt tolerance detection in eighteen wheat genotypes. Plants 9, 287. doi: 10.3390/plants9030287 32106488 PMC7154827

[B4] Albaladejo I. Egea I. Morales B. Flores F. B. Capel C. Lozano R.. (2018). Identification of key genes involved in the phenotypic alterations of *res* (*restored cell structure by salinity*) tomato mutant and its recovery induced by salt stress through transcriptomic analysis. BMC Plant Biol. 18, 1–19. doi: 10.1186/s12870-018-1436-9 30285698 PMC6167845

[B5] Bailey-Serres J. Parker J. E. Ainsworth E. A. Oldroyd G. E. D. Schroeder J. I. (2019). Genetic strategies for improving crop yields. Nature 575, 109–118. doi: 10.1038/s41586-019-1679-0 31695205 PMC7024682

[B6] Baksh S. K. Y. Donde R. Kumar J. Mukherjee M. Meher J. Behera L.. (2021). Genetic relationship, population structure analysis and phenomolecular characterization of rice (*Oryza sativa* L.) cultivars for bacterial leaf blight resistance and submergence tolerance using trait specific STS markers. Physiol. Mol. Biol. Plants 27, 543–562. doi: 10.1007/s12298-021-00951-1 33854283 PMC7981353

[B7] Bhattarai S. Biswas D. Fu Y. Biligetu B. (2020). Morphological, physiological, and genetic responses to salt stress in alfalfa: a review. Agronomy 10, 577. doi: 10.3390/agronomy10040577

[B8] Boonchai C. Udomchalothorn T. Sripinyowanich S. Comai L. Buaboocha T. Chadchawan S. (2018). Rice overexpressing *OsNUC1-S* reveals differential gene expression leading to yield loss reduction after salt stress at the booting stage. Int. J. Mol. Sci. 19, 3936. doi: 10.3390/ijms19123936 30544581 PMC6320848

[B9] Di F. Jian H. Wang T. Chen X. Ding Y. Du H.. (2018). Genome-wide analysis of the *PYL* gene family and identification of *PYL* genes that respond to abiotic stress in *Brassica napus* . Genes 9, 156. doi: 10.3390/genes9030156 29534558 PMC5867877

[B10] Dietz K. Turkan I. Krieger-Liszkay A. (2016). Redox- and reactive oxygen species-dependent signaling into and out of the photosynthesizing chloroplast. Plant Physiol. 171, 1541–1550. doi: 10.1104/pp.16.00375 27255485 PMC4936569

[B11] Egea I. Pineda B. Ortiz-Atienza A. Plasencia F. A. Drevensek S. (2018). The S1CBL10 calcineurin b-like protein ensures plant growth under salt stress by regulating Na^+^ and Ca^2+^ homeostasis. Plant Physiol. 2, 1676–1693. doi: 10.1104/pp.17.01605 PMC5813568 29229696

[B12] Fahad S. Hussain S. Matloob A. Khan F. A. Khaliq A. Saud S.. (2015). Phytohormones and plant responses to salinity stress: a review. Plant Growth Regul. 75, 391–404. doi: 10.1007/s10725-014-0013-y

[B13] Fukuyama K. (2004). Structure and function of plant-type ferredoxins. Photosynth. Res. 81, 289–301. doi: 10.1023/B:PRES.0000036882.19322.0a 16034533

[B14] Ganie S. A. Wani S. H. Henry R. Hensel G. (2021). Improving rice salt tolerance by precision breeding in a new era. Curr. Opin. Plant Biol. 60, 101996. doi: 10.1016/j.pbi.2020.101996 33444976

[B15] Gao C. Li P. Song A. Wang H. Wang Y. Ren L.. (2015). Isolation and characterization of six *AP2/ERF* transcription factor genes in *Chrysanthemum nankingense* . Int. J. Mol. Sci. 16, 2052–2065. doi: 10.3390/ijms16012052 25607731 PMC4307348

[B16] Gong Z. Xiong L. Shi H. Yang S. Herrera-Estrella L. R. Xu G.. (2020). Plant abiotic stress response and nutrient use efficiency. Sci. China Life Sci. 63, 635–674. doi: 10.1007/s11427-020-1683-x 32246404

[B17] Gupta P. Nutan K. K. Singla-Pareek S. L. Pareek A. (2017). Abiotic stresses cause differential regulation of alternative splice forms of GATA transcription factor in rice. Front. Plant Sci. 8. doi: 10.3389/fpls.2017.01944 PMC5693882 29181013

[B18] Hagen G. Guilfoyle T. (2002). Auxin-responsive gene expression: genes, promoters and regulatory factors. Plant Mol. Biol. 49, 373–385. doi: 10.1023/A:1015207114117 12036261

[B19] Hooks T. Masabni J. Ganjegunte G. Sun L. Chandra A. Niu G. (2022). Salt tolerance of seven genotypes of zoysiagrass (*zoysia* spp.). Technol. Horticult. 2, 1–7. doi: 10.48130/TIH-2022-0008

[B20] Jin T. Sun Y. Zhao R. Shan Z. Gai J. Li Y. (2019). Overexpression of peroxidase gene *GsPRX9* confers salt tolerance in soybean. Int. J. Mol. Sci. 20, 3745. doi: 10.3390/ijms20153745 31370221 PMC6695911

[B21] Jung Y. J. Lee I. H. Nou I. S. Lee K. D. Rashotte A. M. Kang K. K. (2013). *BrRZFP1* a *Brassica rapa* C3HC4-type Ring zinc finger protein involved in cold, salt and dehydration stress. Plant Biol. (Stuttg) 15, 274–283. doi: 10.1111/j.1438-8677.2012.00631.x 22726580

[B22] Kang J. Hwang J. Lee M. Kim Y. Assmann S. M. Martinoia E.. (2010). PDR-type ABC transporter mediates cellular uptake of the phytohormone abscisic acid. Proc. Natl. Acad. Sci. 107, 2355–2360. doi: 10.1073/pnas.0909222107 20133880 PMC2836657

[B23] Khan H. A. Siddique K. H. M. Colmer T. D. (2016). Salt sensitivity in chickpea is determined by sodium toxicity. Planta 244, 623–637. doi: 10.1007/s00425-016-2533-3 27114264

[B24] Kotula L. Clode P. L. Jimenez J. D. L. C. Colmer T. D. (2019). Salinity tolerance in chickpea is associated with the ability to ‘exclude’ Na from leaf mesophyll cells. J. Exp. Bot. 70, 4991–5002. doi: 10.1093/jxb/erz241 31106833 PMC6760269

[B25] Kumari P. H. Kumar S. A. Sivan P. Katam R. Suravajhala P. Rao K. S.. (2017). Overexpression of a plasma membrane bound Na^+^/H^+^ antiporter-like protein (*SbNHXLP*) confers salt tolerance and improves fruit yield in tomato by maintaining ion homeostasis. Front. Plant Sci. 7. doi: 10.3389/fpls.2016.02027 PMC5216050 28111589

[B26] Li P. Chai Z. Lin P. Huang C. Huang G. Xu L.. (2020). Genome-wide identification and expression analysis of AP2/ERF transcription factors in sugarcane (*Saccharum spontaneum* L.). BMC Genomics 21, 1–17. doi: 10.1186/s12864-020-07076-x PMC7531145 33008299

[B27] Li Q. Song J. Zhou Y. Chen Y. Zhang L. Pang Y.. (2022). Full-length transcriptomics reveals complex molecular mechanism of salt tolerance in *Bromus inermis* L. Front. Plant Sci. 13. doi: 10.3389/fpls.2022.917338 PMC9219601 35755679

[B28] Li S. Wang Y. Gao X. Lan J. Fu B. (2022). Comparative physiological and transcriptome analysis reveal the molecular mechanism of melatonin in regulating salt tolerance in alfalfa (*Medicago sativa* L.). Front. Plant Sci. 13. doi: 10.3389/fpls.2022.919177 PMC9326453 35909721

[B29] Liang W. Ma X. Wan P. Liu L. (2018). Plant salt-tolerance mechanism: a review. Biochem. Bioph. Res. Co. 495, 286–291. doi: 10.1016/j.bbrc.2017.11.043 29128358

[B30] Liang Q. Y. Wu Y. H. Wang K. Bai Z. Y. Liu Q. L. Pan Y. Z.. (2017). Chrysanthemum WRKY gene *DgWRKY5* enhances tolerance to salt stress in transgenic chrysanthemum. Sci. Rep. 7, 4799. doi: 10.1038/s41598-017-05170-x 28684847 PMC5500475

[B31] Livak K. J. Schmittgen T. D. (2001). Analysis of relative gene expression data using real-time quantitative pcr and the 2^–ΔΔCT^ * ^C^ * ^T^ method. Methods 25, 402–408. doi: 10.1006/meth.2001.1262 11846609

[B32] Lv X. Chen S. Wang Y. (2019). Advances in understanding the physiological and molecular responses of sugar beet to salt stress. Front. Plant Sci. 10. doi: 10.3389/fpls.2019.01431 PMC6851198 31781145

[B33] Ma D. Cai J. Ma Q. Wang W. Zhao L. Li J.. (2022). Comparative time-course transcriptome analysis of two contrasting alfalfa (*Medicago sativa* L.) genotypes reveals tolerance mechanisms to salt stress. Front. Plant Sci. 13. doi: 10.3389/fpls.2022.1070846 PMC9773191 36570949

[B34] Matsuoka D. Soga K. Yasufuku T. Nanmori T. (2018). Control of plant growth and development by overexpressing *MAP3K17*, an ABA-inducible MAP3K, in *Arabidopsis* . Plant Biotechnol. Nar. 35, 171–176. doi: 10.5511/plantbiotechnology.18.0412a PMC6879389 31819720

[B35] Morgil H. Tardu M. Cevahir G. Kavakli I. H. (2019). Comparative RNA-seq analysis of the drought-sensitive lentil (*Lens culinaris*) root and leaf under short- and long-term water deficits. Funct. Integr. Genomic. 19, 715–727. doi: 10.1007/s10142-019-00675-2 31001704

[B36] Mudalkar S. Sreeharsha R. V. Reddy A. R. (2017). Involvement of glyoxalases and glutathione reductase in conferring abiotic stress tolerance to *Jatropha curcas* L. Environ. Exp. Bot. 134, 141–150. doi: 10.1016/j.envexpbot.2016.11.011

[B37] Nguyen V. N. T. Moon S. Jung K. (2014). Genome-wide expression analysis of rice ABC transporter family across spatio-temporal samples and in response to abiotic stresses. J. Plant Physiol. 171, 1276–1288. doi: 10.1016/j.jplph.2014.05.006 25014263

[B38] Shani E. Salehin M. Zhang Y. Sanchez S. E. Doherty C. Wang R.. (2017). Plant stress tolerance requires Auxin-sensitive Aux/IAA transcriptional repressors. Curr. Biol. 27, 437–444. doi: 10.1016/j.cub.2016.12.016 28111153 PMC5296222

[B39] Sharifi P. (2010). Evaluation on sixty-eight rice germplasms in cold tolerance at germination stage. Rice Sci. 17, 77–81. doi: 10.1016/S1672-6308(08)60107-9

[B40] Sharma R. Sahoo A. Devendran R. Jain M. (2014). Over-expression of a rice tau class glutathione s-transferase gene improves tolerance to salinity and oxidative stresses in Arabidopsis: e92900. PloS One 9, e92900. doi: 10.1371/journal.pone.0092900 24663444 PMC3963979

[B41] Steinhorst L. Kudla J. (2019). How plants perceive salt. Nature 572, 318–320. doi: 10.1038/d41586-019-02289-x 31406308

[B42] Sukiran N. A. Steel P. G. Knight M. R. (2020). Basal stomatal aperture is regulated by GA-DELLAs in arabidopsis. J. Plant Physiol. 250, 153182. doi: 10.1016/j.jplph.2020.153182 32428693

[B43] Sun S. J. Guo S. Q. Yang X. Bao Y. M. Tang H. J. Sun H.. (2010). Functional analysis of a novel Cys2/His2-type zinc finger protein involved in salt tolerance in rice. J. Exp. Bot. 61, 2807–2818. doi: 10.1093/jxb/erq120 20460361 PMC2882275

[B44] Sun K. Mehari T. G. Fang H. Han J. Huo X. Zhang J.. (2023). Transcriptome, proteome and functional characterization reveals salt stress tolerance mechanisms in upland cotton (*Gossypium hirsutum* L.). Front. Plant Sci. 14. doi: 10.3389/fpls.2023.1092616 PMC9978342 36875590

[B45] Trivedi D. K. Gill S. S. Yadav S. Tuteja N. (2013). Genome-wide analysis of glutathione reductase (GR) genes from rice and Arabidopsis. Plant Signal Behav. 8, e23021. doi: 10.4161/psb.23021 23221779 PMC3657001

[B46] Van Bezouw R. F. H. M. Janssen E. M. Ashrafuzzaman M. Ghahramanzadeh R. Kilian B. Graner A.. (2019). Shoot sodium exclusion in salt stressed barley (*Hordeum vulgare* L.) is determined by allele specific increased expression of *HKT1; 5* . J. Plant Physiol. 241, 153029. doi: 10.1016/j.jplph.2019.153029 31499444

[B47] Vani B. Saradhi P. Mohanty P. (2001). Alteration in chloroplast structure and thylakoid membrane composition due to invivoheat treatment of rice seedlings: correlation with the functional changes. J. Plant Physiol. 158, 583–592. doi: 10.1078/0176-1617-00260

[B48] Wang H. Chen W. Xu Z. Chen M. Yu D. (2023). Functions of WRKYs in plant growth and development. Trends Plant Sci. 28, 630–645. doi: 10.1016/j.tplants.2022.12.012 36628655

[B49] Wang Y. Li Y. (2013). Land exploitation resulting in soil salinization in a desert-oasis ecotone. Catena 100, 50–56. doi: 10.1016/j.catena.2012.08.005

[B50] Wang X. Li X. Cai D. Lou J. Li D. Liu F. (2021). Salinification and salt transports under aeolian processes in potential desertification regions of China. Sci. Total Environ. 782, 146832. doi: 10.1016/j.scitotenv.2021.146832

[B51] Wang W. Liu Y. Duan H. Yin X. Cui Y. Chai W.. (2020). Sshkt1; 1 is coordinated with sssos1 and ssnhx1 to regulate Na^+^ homeostasis in *Suaeda salsa* under saline conditions. Plant Soil 449, 117–131. doi: 10.1007/s11104-020-04463-x

[B52] Wu W. Zhang Q. Ervin E. H. Yang Z. Zhang X. (2017). Physiological mechanism of enhancing salt stress tolerance of perennial ryegrass by 24-epibrassinolide. Front. Plant Sci. 8. doi: 10.3389/fpls.2017.01017 PMC5474491 28674542

[B53] Yang Y. Gao S. Su Y. Lin Z. Guo J. Li M.. (2019). Transcripts and low nitrogen tolerance: regulatory and metabolic pathways in sugarcane under low nitrogen stress. Environ. Exp. Bot. 163, 97–111. doi: 10.1016/j.envexpbot.2019.04.010

[B54] Yin Y. L. Xu Y. N. Li X. N. Fan S. G. Wang G. Y. Fu J. M.. (2022). Physiological integration between Bermudagrass ramets improves overall salt resistance under heterogeneous salt stress. Physiol. Plantarum 174, e13655. doi: 10.1111/ppl.13655 35243634

[B55] Yu Z. Duan X. Luo L. Dai S. Ding Z. Xia G. (2020). How plant hormones mediate salt stress responses. Trends Plant Sci. 25, 1117–1130. doi: 10.1016/j.tplants.2020.06.008 32675014

[B56] Yu S. Wu J. Wang M. Shi W. Xia G. Jia J.. (2020). Haplotype variations in QTL for salt tolerance in chinese wheat accessions identified by marker-based and pedigree-based kinship analyses. Crop J. 8, 1011–1024. doi: 10.1016/j.cj.2020.03.007

[B57] Zhang M. Bai R. Nan M. Ren W. Wang C. Shabala S.. (2022). Evaluation of salt tolerance of oat cultivars and the mechanism of adaptation to salinity +. J. Plant Physiol. 273, 153708. doi: 10.1016/j.jplph.2022.153708 35504119

[B58] Zhang G. Chen M. Li L. Xu Z. Chen X. Guo J.. (2009). Overexpression of the soybean *GmERF3* gene, an AP2/ERF type transcription factor for increased tolerances to salt, drought, and diseases in transgenic tobacco. J. Exp. Bot. 60, 3781–3796. doi: 10.1093/jxb/erp214 19602544 PMC2736888

[B59] Zhang J. Shi H. (2013). Physiological and molecular mechanisms of plant salt tolerance. Photosynth. Res. 115, 1–22. doi: 10.1007/s11120-013-9813-6 23539361

[B60] Zhang L. Sun Y. Ji J. Zhao W. Guo W. Li J.. (2023). Flavonol synthase gene *MsFLS13* regulates saline-alkali stress tolerance in alfalfa. Crop J. 11, 1218–1229. doi: 10.1016/j.cj.2023.05.003

[B61] Zhao Y. Zhang F. Mickan B. Wang D. Wang W. (2022). Physiological, proteomic, and metabolomic analysis provide insights into *Bacillus* sp. -Mediated salt tolerance in wheat. Plant Cell Rep. 41, 95–118. doi: 10.1007/s00299-021-02788-0 34546426

[B62] Zhao C. Zhang H. Song C. Zhu J. K. Shabala S. (2020). Mechanisms of plant responses and adaptation to soil salinity. Innovation (Camb) 1, 100017. doi: 10.1016/j.xinn.2020.100017 34557705 PMC8454569

[B63] Zhou X. Wang F. Ma Y. Jia L. Liu N. Wang H.. (2018). Ectopic expression of SsPETE2, a plastocyanin from *Suaeda salsa*, improves plant tolerance to oxidative stress. Plant Sci. 268, 1–10. doi: 10.1016/j.plantsci.2017.12.006 29362078

[B64] Zhu J. (2016). Abiotic stress signaling and responses in plants. Cell 167, 313–324. doi: 10.1016/j.cell.2016.08.029 27716505 PMC5104190

[B65] Zhu J. Wei X. Yin C. Zhou H. Yan J. He W.. (2023). ZmEREB57 regulates OPDA synthesis and enhances salt stress tolerance through two distinct signalling pathways in *Zea mays* . Plant Cell Environ. 46, 2867–2883. doi: 10.1111/pce.14644 37326336

