# Supplementary material for: Transcriptome analysis and physiological changes in the leaves of two Bromus inermis L. genotypes in response to salt stress
Source: Front Plant Sci. 2023 Dec 14;14:1313113. doi: 10.3389/fpls.2023.1313113 (PMC10755925; doi:10.3389/fpls.2023.1313113)
Supplement: Supplementary file 1 [file DataSheet_1.docx]

Supplementary Figure 1. Frequency distributions of growth traits of 57 smooth bromegrass materials under Control (red) or NaCl (blue).

Supplementary Figure 2. Pearson correlation figures among each traits relative value across 57 smooth bromegrass accessions under NaCl treatment. Figure 2-Ⅰ. A: Germination potential; B: Germination rate; C: Radicle length; D: Embryo length; E: Root bud ratio; F: Germination index; G: Vitality index. Figure 2-Ⅱ. A: Plant height; B: Leaf length; C: Leaf width; D: Leaf area; E: Fresh weight above ground; F: Fresh weight under ground; G: Dry weight above ground; H: Dry weight under ground; I: Root to shoot ratio; J: Root length; K: Root project area; L: Root surface area; M: Root average diameter; N: Root volume; O: Root tips. * and ** mean significant correlation at 0.05 and 0.01 level, respectively.

Supplementary Figure 3. Macadam diagram of principal component analysis.

Supplementary Figure 4. Principal component profile. A: Plant height; B: Leaf length; C: Leaf width; D: Leaf area; E: Fresh weight above ground; F: Fresh weight under ground; G: Dry weight above ground; H: Dry weight under ground; I: Root to shoot ratio; J: Root length; K: Root project area; L: Root surface area; M: Root volume; N: Root tips; O: Germination potential; P: Germination rate; Q: Radicle length; R: Embryo length; S: Germination index; T: Vitality index.


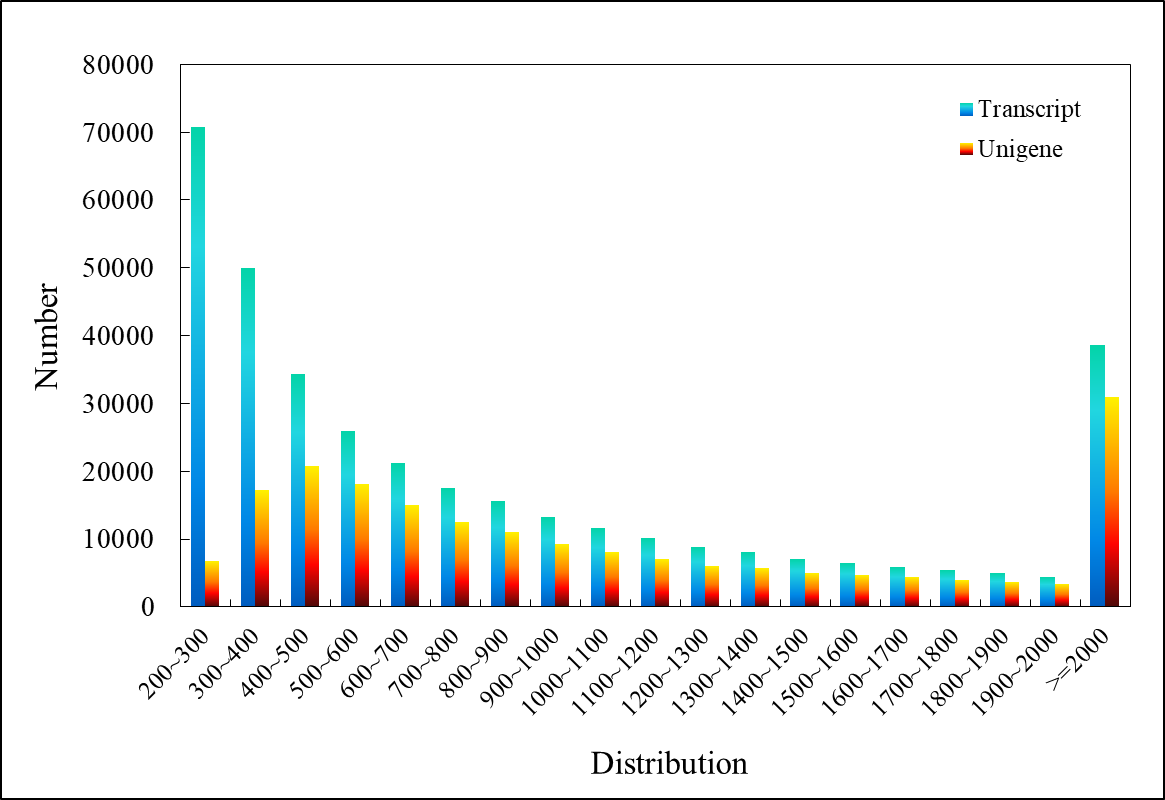


Supplementary Figure 5. Sequencing length distribution of unigenes and transcripts of smooth bromegrass seedlings under salt stress.


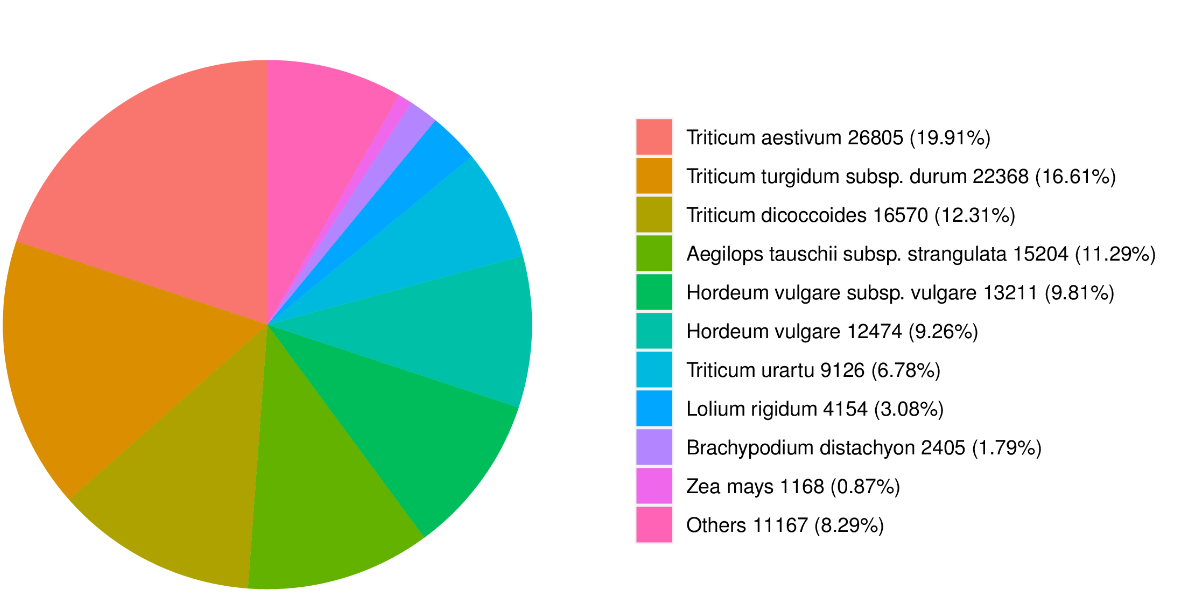


Supplementary Figure 6. Species distribution map of smooth bromegrass in Nr database alignment.


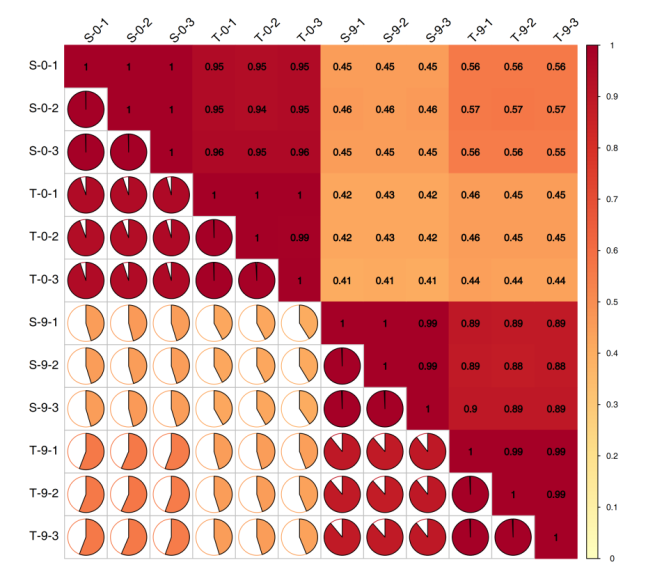


Supplementary Figure 7. Correlation test of samples with different treatments in smooth bromegrass.


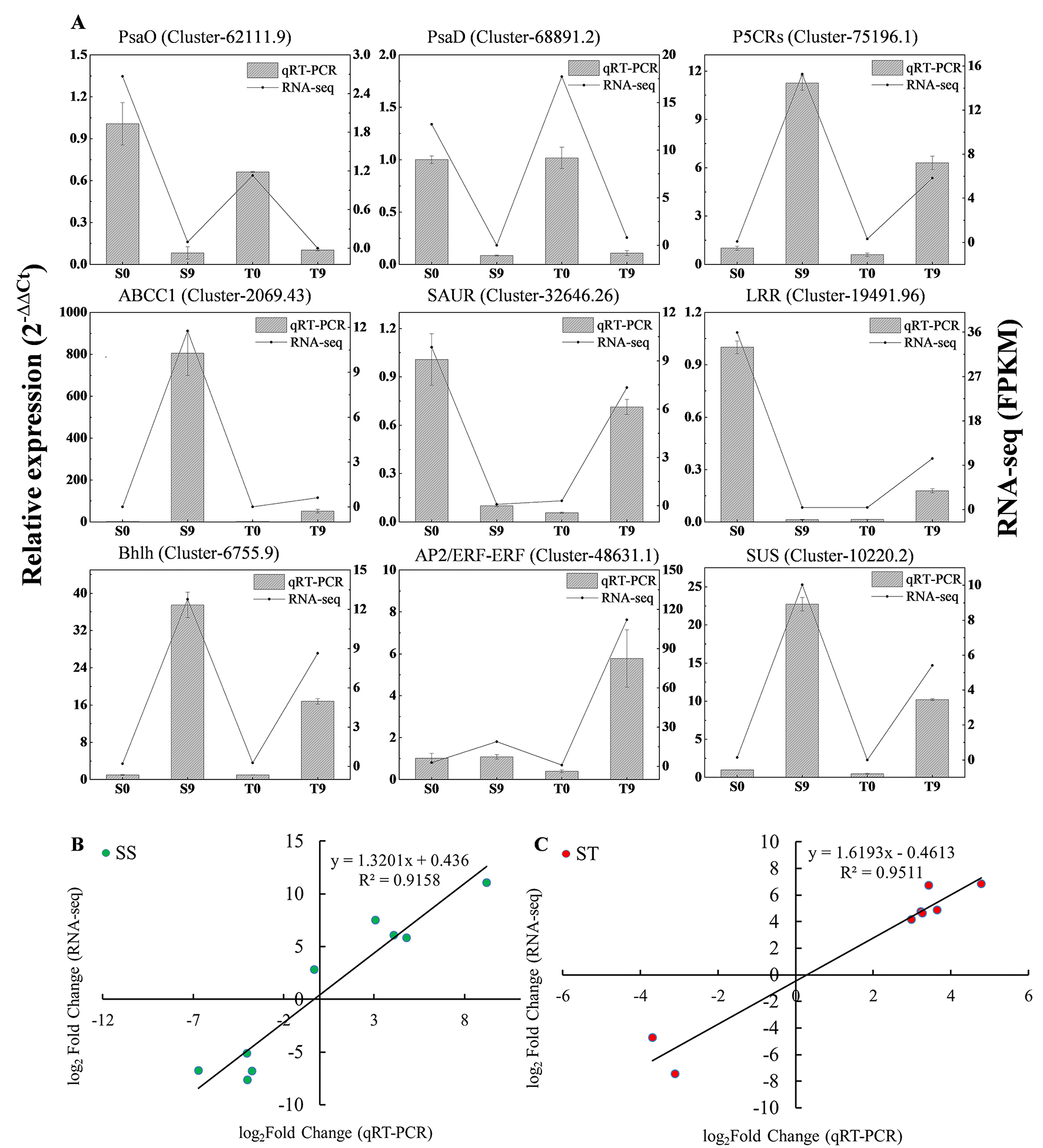


Supplementary Figure 8. qRT-PCR validation of expression profiles obtained by RNA-Seq in SS and ST under salt stress. A. qRT-PCR and RNA-seq analysis of 9 genes. The correlation of log_2_Fold Change obtained by qRT-PCR (X-axis) and RNA-seq (Y-axis) in SS (B) and ST (C). SS: Salt-sensitive material; ST: Salt-tolerant material.
